# Current status and future perspectives of multi‐modal bacteria‐based cancer therapies

**DOI:** 10.1002/ctm2.70485

**Published:** 2025-10-21

**Authors:** Shuai Fan, Siyu Zhu, Wenyu Wang, Yuetong Liu, Yutong Zhou, Hao Li, Bofeng Liu, Qin Xia, Lili Huang, Lei Dong

**Affiliations:** ^1^ Advanced Technology Research Institute, State Key Laboratory of Molecular Medicine and Biological Diagnosis and Treatment (Ministry of Industry and Information Technology), School of Life Science Beijing Institute of Technology Beijing China; ^2^ School of Medical Technology Beijing Institute of Technology Beijing China; ^3^ China Talent Group Beijing China

**Keywords:** bacteria, cancer, TME, genetic modification, nanotechnology, synergistic therapy

## Abstract

**Background:**

Targeted drug delivery systems have garnered increasing research interest in cancer threapy. Bacteria have emerged as a promising vehicle due to their innate ability to the tumour microenvironment (TME) and their intrinsic immune‐stimulating properties. This review explores the application of bacteria in oncology, emphasizing the tumour‐targeting behaviour of specific strains, their immunomodulatory functions, and their potential as delivery platforms for the controlled release of therapeutic agents.

**Main text:**

This review synthesizes recent advances in bacteria‐mediated cancer therapy, focusing on the mechanisms underlying bacterial targeting of hypoxic and immunosuppressive regions within the tumor microenvironment (TME). We discuss how genetic modification has been employed to design recombinant bacterial strains with enhanced tumor specificity and amplified therapeutic effects. Furthermore, the integration of bacteria with nanotechnology has facilitated the development of hybrid systems capable of targeted drug delivery and triggered‐release mechanisms. The combination of bacterial therapy with other treatment modalities—such as photodynamic (PDT) and sonodynamic therapies (SDT)—is also examined, emphasizing their synergistic potential in overcoming tumor heterogeneity and enhancing anti‐tumor immunity. Finally, we survey the current clinical progress of bacteria‐based therapeutics and offer perspectives on the future role of artificial intelligence (AI) in improving the design and application of these living medicines.

**Conclusions:**

Bacteria‐based delivery systems represent a multifunctional and innovative strategy in the evolution of targeted cancer therapies. Through genetic modification and nanobiotechnology approaches, bacteria can be customized to mediate multi‐effect synergistic treatments for cancer, enhancing the precision, safety, and efficacy of cancer therapies. With the ongoing integration of advanced technologies, including AI, there is great potential to overcome existing limitations and accelerate the clinical translation of bacterial anticancer therapies. This interdisciplinary effort is poised to open new avenues for next‐generation cancer treatments and lay the foundation for future directions in cancer research and therapeutic practice.

**Key points:**

- Bacteria exhibit inherent tumour‐targeting capabilities, particularly thriving in hypoxic tumour microenvironments (TMEs) and activating potent anti‐tumour immune responses through pathogen‐associated molecular patterns (PAMPs) and immunomodulation.
- Genetic engineering and nanobiotechnology enable advanced bacterial therapies, allowing for reduced toxicity, controlled proliferation, targeted drug delivery and the expression of therapeutic payloads (e.g., cytokines, enzymes, tumour antigens) within tumours.
- Bacteria serve as versatile platforms for multi‐modal synergistic therapy, effectively combining with immunotherapy, photodynamic therapy (PDT), thermodynamic therapy (TDT), photothermal therapy (PTT) and sonodynamic therapy (SDT) to significantly enhance tumour eradication.
- Artificial intelligence (AI) is poised to revolutionise bacterial cancer therapy, offering powerful tools for optimising synthetic biology designs (e.g., promoters, gene circuits), nanocarrier engineering and predicting bacterial–host interactions for more effective and safer treatments.

## Graphical abstract

1. Bacteria exhibit inherent tumour‐targeting capabilities, particularly thriving in hypoxic tumour microenvironments (TMEs) and activating potent anti‐tumour immune responses through pathogen‐associated molecular patterns (PAMPs) and immunomodulation.
2. Genetic engineering and nanobiotechnology enable advanced bacterial therapies, allowing for reduced toxicity, controlled proliferation, targeted drug delivery and the expression of therapeutic payloads (e.g., cytokines, enzymes, tumour antigens) within tumours.
3. Bacteria serve as versatile platforms for multi‐modal synergistic therapy, effectively combining with immunotherapy, photodynamic therapy (PDT), thermodynamic therapy (TDT), photothermal therapy (PTT) and sonodynamic therapy (SDT) to significantly enhance tumour eradication.
4. Artificial intelligence (AI) is poised to revolutionise bacterial cancer therapy, offering powerful tools for optimising synthetic biology designs (e.g., promoters, gene circuits), nanocarrier engineering and predicting bacterial–host interactions for more effective and safer treatments.

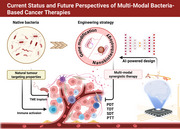

## 1 INTRODUCTION

Cancer is a major disease that severely threatens human health.^1^ According to recent estimates, the global cancer burden is expected to increase significantly in the coming decades (by 47% from 2023 to 2040).^2^ Traditional cancer treatments, such as chemotherapy and radiotherapy, are often limited by their systemic toxicity and non‐specific targeting, resulting in serious side effects and damage to healthy tissues.^3^, ^4^ In recent years, with the ongoing advancements of nanotechnology and gene editing, bacteria‐based cancer therapies have emerged as a promising approach. These therapies leverage the unique characteristics of bacteria to specifically target the tumour microenvironment (TME) and mediate the destruction of cancer cells, offering effective solutions for the treatment of solid tumours such as bladder cancer, colorectal cancer and pancreatic cancer.^5^, ^6^, ^7^, ^8^ The use of bacteria‐based active materials for precise cancer diagnosis and treatment has become a research hotspot.^9^ Possessing key nanomaterial‐like properties such as biofilm formation and highly modifiable cell walls, bacteria exhibit excellent tumour targeting, biocompatibility and controllability, showing tremendous potential in tumour therapy.^10^ Bacterial therapies offer strong TME targeting, with lower toxicity and fewer side effects compared to conventional therapies.^11^

Using bacteria for cancer therapy is not a new concept. As early as the late 19th century, William Coley observed that bacterial infections could lead to tumour regression.^12^ Since then, increasing evidence has shown that certain bacteria exhibit strong anti‐tumour effects and can target the TME of solid tumours. Engineered bacteria, such as *Clostridium novyi*‐NT (*C. novyi‐*NT) and attenuated *Salmonella enterica* serovar Typhimurium (*S*. Typhimurium) strains, have demonstrated potential in targeting hypoxic tumour regions and inducing potent anti‐tumour immune responses.^13^, ^14^ The tumour‐targeting ability of bacteria is primarily attributed to the unique characteristics of the TME, including hypoxia, low pH and immune suppression.^11^, ^15^, ^16^ These environmental conditions create favourable conditions for the growth and proliferation of specific bacteria, allowing them to selectively colonise tumour tissues. However, several critical technological gaps must be addressed to translate this colonisation potential into viable bacterial therapies. First, the precise spatiotemporal control of bacterial proliferation within tumours remains elusive—unregulated overgrowth could trigger systemic toxicity or disrupt the TME homeostasis, while insufficient colonisation limits therapeutic efficacy. Second, the mechanisms underlying bacterial evasion of host immune surveillance in the TME are incompletely understood; without strategies to shield bacteria from phagocytosis by tumour‐associated neutrophils or clearance by other immune cells, their persistence and functional duration are severely constrained. Third, the specificity of bacterial targeting to tumours versus normal tissues requires enhancement, as off‐target colonisation in healthy organs may induce unwanted inflammation or infection. The coordination of bacterial effector functions (e.g., cytokine secretion, oncolysis) with the dynamic TME, including adaptability to fluctuating oxygen levels, nutrient gradients and immune cell infiltration, remains poorly optimised. These gaps underscore the necessity for engineered bacterial strains with tuneable growth dynamics, immune‐evasive properties and context‐responsive effector modules, making such engineering efforts not merely innovative but indispensable for advancing bacterial therapies from preclinical promise to clinical utility.

In recent years, the rapid development of genetic engineering, synthetic biology and nanobiotechnology has reignited the advancement of bacterium‐based cancer therapies, making the development of novel anti‐tumour drugs using bacteria a viable approach.^17^, ^18^ Bacteria‐based therapies primarily focus on two main directions: first, modifying bacteria with nanoparticles to enhance their targeting ability to the TME, allowing for the targeted delivery of chemotherapy drugs, photosensitisers, photothermal agents and other therapeutic agents to precisely deliver them to tumour tissues for effective tumour destruction; second, engineering bacterial chassis strains to reduce bacterial toxicity, enabling the expression of specific tumour‐targeting peptides, receptors or signalling molecules that can target and kill tumour cells.^19^ This approach also involves activating the immune system through bacteria to enhance the body's immune response against the tumour, thereby inhibiting tumour growth. Furthermore, combining bacterial therapy with other modalities, such as photodynamic therapy (PDT), thermodynamic therapy (TDT), sonodynamic therapy (SDT) and radiotherapy, can result in synergistic effects, significantly improving therapeutic outcomes.^20^

This review summarises the principles of bacteria‐based cancer therapy, explores the applications of genetic modification and nanotechnology in this field and provides a detailed overview of bacterial therapies that have progressed to clinical stages. Finally, the review presents a comprehensive discussion of multi‐modal synergistic treatment strategies based on bacteria, emphasising the necessity and potential advantages of combining various therapeutic approaches, and explores the future applications of artificial intelligence (AI) in this field. By integrating immunotherapy, photothermal therapy (PTT) and other treatment modalities, bacteria‐based cancer therapies can achieve a synergistic therapeutic effect that targets multiple hallmarks of tumour progression, thereby enhancing treatment efficacy while minimising treatment‐related side effects.

## 2 BACTERIA IN TUMOUR THERAPY: NATURAL PROPERTIES FOR PRECISION TARGETING AND MULTIFUNCTIONAL REGULATION

**FIGURE 1:**
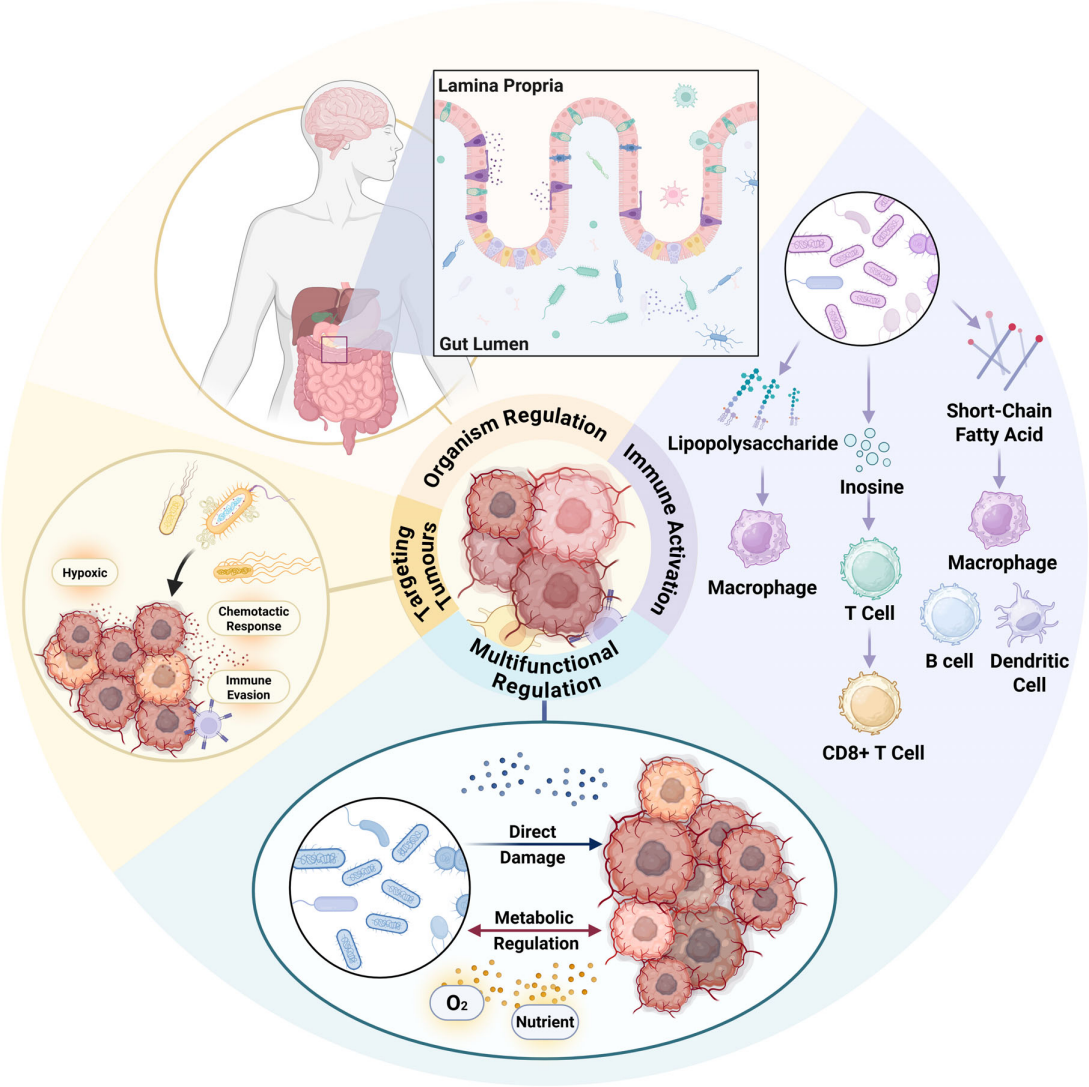
Elucidating the mechanisms underlying bacterial colonisation and anti‐tumour activity. Bacteria target hypoxic tumour microenvironment (TME) through mechanisms including hypoxia‐selective enrichment, chemotaxis and immune evasion. The gut microbiota influences cancer through indirect pathways, such as activating the immune system via molecules like lipopolysaccharides (LPS), inosine and short‐chain fatty acids, which interact with macrophages, T‐cells, B‐cells and dendritic cells. Additionally, bacteria can directly damage tumour cells and regulate their metabolism through interactions with oxygen and nutrients. Created with https://www.biorender.com.

Bacteria exhibit intrinsic properties that uniquely position them as effective agents for tumour therapy. Unlike radiotherapy, oxygen‐dependent chemotherapies, and immunotherapies—whose efficacy is often compromised in hypoxic TMEs due to impaired drug penetration, reduced reactive oxygen species (ROS) generation and immunosuppressive microenvironments—bacteria thrive under hypoxia. Their ability to actively target and colonise hypoxic niches,^21^ coupled with innate immune‐modulating capabilities, enables precise spatiotemporal regulation of tumour growth and microenvironment dynamics.^22^ These inherent traits, amplified by genetic and metabolic engineering, underscore bacteria's promise as versatile platforms for next‐generation cancer therapies (Figure 1).

### 2.1 Targeting tumours: Natural ‘navigators’ in hypoxic microenvironments

**FIGURE 2:**
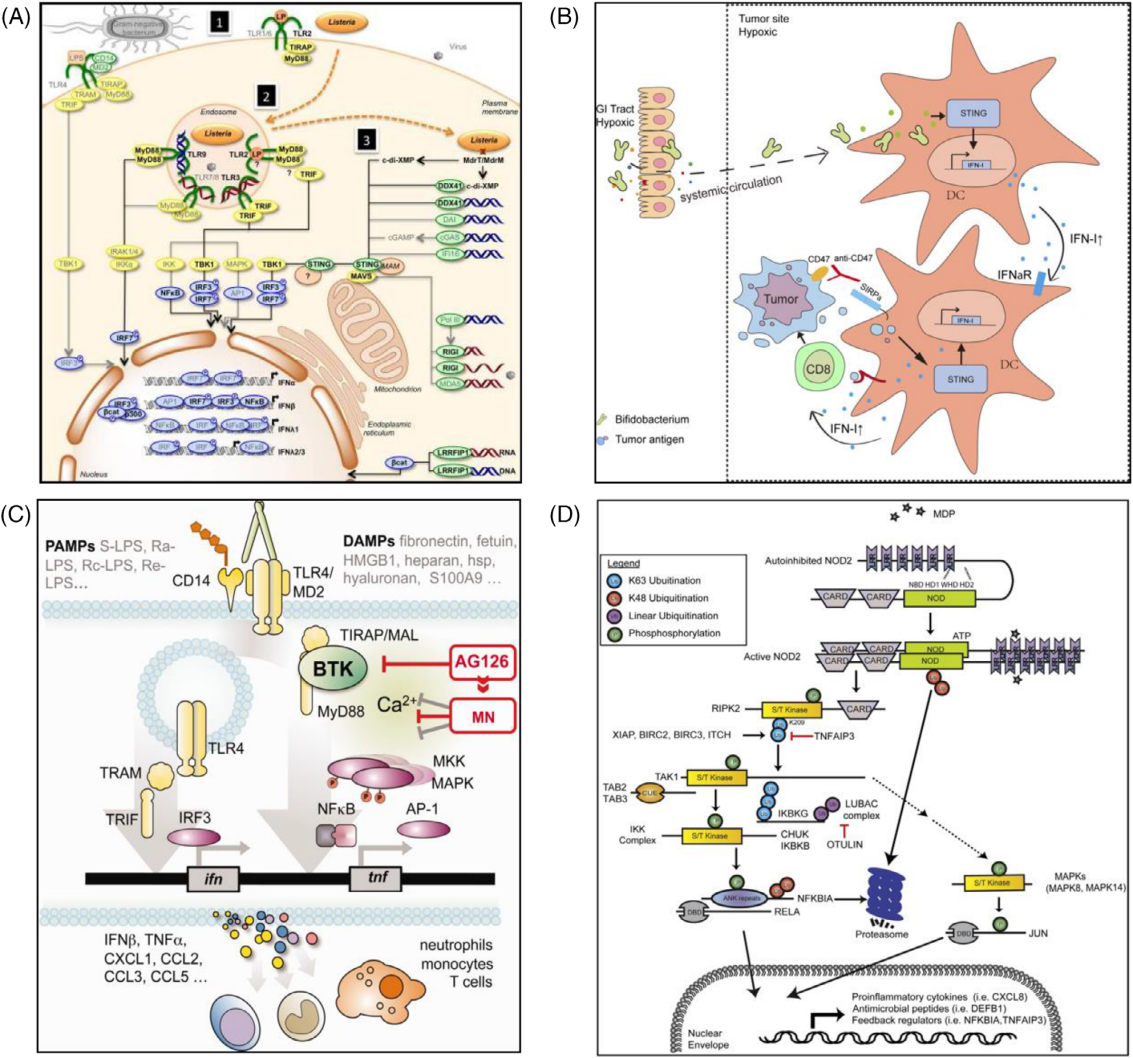
Molecular mechanisms of bacterial tumour targeting and immunomodulation. (A) Activation of type I‐ and type III‐IFNs by infection. Adapted from ref., 42 with permission of Frontiers Media S.A., copyright (2014). (B) Enhancing the immune response mediated by CD47 blockade through the stimulation of the STING and interferon‐dependent mechanisms. Adapted from ref., 47 with permission of Rockefeller University Press, copyright (2020). (C) TLR4‐agonistic pathogen‐associated molecular patterns (PAMPs) or damage‐ associated molecular patterns (DAMPs) can trigger in cells, such as microglia, the induction of pro‐inflammatory cytokines/chemokines for recruitment of immune cells. Adapted from ref., ^48^ with permission of John Wiley and Sons, copyright (2015). (D) NOD2 interacts directly with intracellular bacterial PGN fragments containing the MDP motif. Adapted from ref., 49 with permission of Elsevier, copyright (2014).

The hypoxic TME represents a hallmark of solid tumours, arising from an imbalance between rapid tumour cell proliferation and aberrant angiogenesis.^23^, ^24^ Under hypoxic conditions (oxygen concentration typically 1%–4%), tumour cells activate hypoxia‐inducible factor (HIF‐1α/2α) signalling pathways, driving metabolic reprogramming (e.g., the Warburg effect) and angiogenesis, while inducing the expression of immunosuppressive molecules, collectively promoting tumour progression.^25^ Notably, this unique physiological environment provides natural ‘navigational’ signals for certain anaerobic or facultative anaerobic bacteria.^26^, ^27^, ^28^ Research has demonstrated that natural bacteria target the hypoxic TME through several key mechanisms:

1. *Hypoxia‐selective enrichment*: Anaerobic bacteria (e.g., *Clostridium*) and facultative anaerobic bacteria (e.g., *S*. Typhimurium) can sense and preferentially colonise necrotic or hypoxic regions of tumours.^29^, ^30^, ^31^, ^32^ In the TME, the abnormal structure and function of tumour blood vessels lead to the formation of hypoxic and necrotic regions within the tumour, characterised by low oxygen concentrations and low pH. These conditions provide an ideal environment for strict anaerobes and facultative anaerobes to thrive.^33^, ^34^ For instance, *Clostridium* spores germinate exclusively under hypoxic conditions, while their activity is suppressed in the oxygen‐rich environment of normal tissues, achieving tumour‐specific localisation.^35^, ^36^, ^37^
2. *Chemotactic response*: Chemical attractants released by necrotic tumour regions (e.g., aspartate, serine, ribose) can guide bacterial migration towards tumours.^38^ For example, *Salmonella* utilises specific chemoreceptors to recognise metabolites in necrotic tissues and relies on flagellar motility to penetrate the tumour matrix, reaching deep into the hypoxic core.^39^, ^40^
3. *Immune evasion support*: The immunosuppressive properties of the TME (e.g., regulatory T‐cell infiltration, PD‐L1 upregulation) protect tumour cells and concurrently create a ‘safe haven’ for bacteria. Initially, bacteria may enter tumours by passive entrapment within the disordered tumour vasculature, followed by infiltration into the tumour due to inflammation caused by a sudden increase in tumour necrosis factor‐alpha (TNF‐α) in the tumour blood vessels.^15^ *Listeria monocytogenes* (*Lm*), a Gram‐positive intracellular pathogen, is typically associated with foodborne illnesses.^41^ *Lm* suppresses the Th1 immune response driven by IL‐12 and interferon (IFN)‐γ through activation of the host type I interferon (IFN‐I) signalling pathway, thereby impairing antibacterial immune surveillance and enabling its persistent proliferation within the immunosuppressive TME (Figure 2A).^42^, ^43^ Preclinical studies have highlighted the tumour‐targeting potential of various natural bacteria. For instance, non‐pathogenic *C. novyi‐NT* selectively colonises necrotic regions of murine tumours after injection, secreting enzymes to directly lyse tumour cells and inducing local inflammatory responses to enhance anti‐tumour effects.^44^, ^45^, ^46^

In summary, natural bacteria achieve precise TME navigation by integrating hypoxia sensing, chemotactic migration and immune evasion mechanisms, thereby establishing a theoretical foundation for Bacteria‐based anti‐tumour strategies.^50^

### 2.2 An essential component of the human body: The important gut microbiota

A substantial body of research has demonstrated that microorganisms in the body play a crucial role in combating tumours, with the majority of these microorganisms being primarily distributed in the gut.^51^ Gut microbiota plays a pivotal role in anti‐tumour immune responses by influencing both innate and adaptive immune systems.^52^, ^53^ Lin et al. identify a new *Hominenteromicrobium* strain YB328 from faeces of patients responding to programmed cell death‐1 (PD‐1) blockade, which activates tumour‐specific CD8+ T‐cells by stimulating CD103^+^CD11b^−^ conventional dendritic cells (cDCs) in the gut to migrate to the TME, enhances the anti‐tumour efficacy of PD‐1 blockade in multiple mouse models, and shows that patients with elevated YB328 abundance have increased infiltration of CD103^+^CD11b^−^ cDCs in tumours and favourable responses to PD‐1 blockade therapy in various cancer types.^54^ Disruption of the gut microbiota has been shown to reduce the efficacy of CpG‐oligonucleotide immunotherapy and platinum‐based chemotherapeutic agents, such as oxaliplatin. In antibiotic‐treated or germ‐free mice, tumour‐infiltrating myeloid cells exhibit diminished responses to therapy, resulting in reduced cytokine production and tumour necrosis.^55^ Cyclophosphamide alters the small intestinal microbiota, inducing translocation of select Gram‐positive bacteria to secondary lymphoid organs, where they drive the differentiation of ‘pathogenic’ T helper 17 (pTH17) cells and enhance memory TH1 responses. Germ‐free or antibiotic‐treated mice exhibit impaired pTH17 activation and diminished cyclophosphamide efficacy, revealing a critical role for Gram‐positive gut bacteria in mediating the drug's anti‐tumour immunity.^56^

The gut microbiome critically modulates the efficacy of anti‐PD‐1 immunotherapy in melanoma patients. Meta‐genomic sequencing reveals that responders demonstrate significantly higher gut microbiome α‐diversity (Shannon index) compared to non‐responders. Specifically, responders exhibit marked enrichment of commensal bacteria from the *Clostridiales* order, particularly *Faecalibacterium prausnitzii*, which correlates with prolonged progression‐free survival (PFS). Conversely, non‐responders show increased abundance of *Bacteroidales* species. Faecal microbiota transplantation from responders into germ‐free mice recapitulates improved tumour control upon PD‐1 blockade, confirming causality. This indicates that the gut microbiome may modulate responses to anti–PD‐1 immunotherapy in melanoma patients.^57^ Sequencing of 16S ribosomal RNA further identifies a correlation between *Bifidobacterium* and anti‐tumour activity. Oral administration of *Bifidobacterium* alone significantly improves tumour control, achieving results comparable to those of PD‐L1‐specific antibody therapy (immune checkpoint blockade [ICB]). Combined treatment almost completely abrogates tumour growth. This enhanced anti‐tumour effect is mediated by the increased activation and accumulation of CD8+ T‐cells within the TME, which is driven by enhanced dendritic cell functionality.^58^

Changes in the gut microbiota can enhance the anti‐tumour efficacy of metastatic T‐cells. Total body irradiation (TBI) disrupts the intestinal mucosal barrier, leading to microbial translocation and systemic release of LPS. LPS binds to liver‐synthesised LPS‐binding protein (LBP), which facilitates its delivery via the CD14 receptor to the TLR4/MD2 complex. This activates the NF‐κB pathway and promotes the secretion of pro‐inflammatory cytokines (e.g., IL‐6). Concurrently, this process eliminates immunosuppressive regulatory T‐cells (Tregs) while enhancing the expression of co‐stimulatory molecules (e.g., CD80/86) on antigen‐presenting cells (APCs). These combined effects augment the expansion and functional efficacy of adoptively transferred tumour‐reactive T‐cells. Thus, TBI converts microbial‐derived signals into enhanced anti‐tumour immunity through the LBP/CD14–TLR4 axis.^59^

Certain gut bacteria play pivotal roles in activating and amplifying anti‐tumour immune responses, significantly influencing the efficacy of tumour immunotherapy through the modulation of various molecular pathways. Some gut commensal microbes accumulate at tumour sites and enhance immune responses mediated by CD47 blockade through a stimulator of interferon genes (STING)‐ and interferon‐dependent mechanism, thereby improving therapeutic outcomes (Figure 2B).^47^ Specific gut bacteria, such as *Helicobacter hepaticus* (*H. hepaticus*), can promote anti‐tumour immune responses in colorectal cancer. Colonisation of the gut by *H. hepaticus* increases the number of intratumoural lymphocytes, particularly CD4+ T‐cells, B‐cells and natural killer (NK) cells, thereby enhancing the anti‐tumour capacity of immune cells. Additionally, *H. hepaticus* colonisation promotes the formation of tertiary lymphoid structures around tumours, which are critical for the activation and differentiation of anti‐tumour immune cells.^60^

Specific gut bacteria modulate PD‐L2 and RGMb expression and disrupt their interactions,^47^ overcoming resistance to PD‐1 blockade in otherwise non‐responsive tumours. This discovery establishes the PD‐L2–RGMb axis as a critical immune evasion mechanism and highlights microbiome‐mediated regulation as a potential therapeutic strategy for patients resistant to current PD‐1 immunotherapy.^61^ These findings underscore the importance of the gut microbiota in regulating anti‐tumour immune responses and suggest the potential of manipulating the gut microbiome.

### 2.3 Immune activation: Bacteria as natural ‘immunopotentiators’

Bacteria activate innate immune responses through the interaction of their natural surface molecules with host pattern recognition receptors (PRRs), a process independent of any genetic engineering and based on evolutionarily conserved pathogen‐associated molecular patterns (PAMPs).^62^ LPS binds to LBP in circulation, which transfers LPS to CD14. CD14 facilitates the loading of LPS onto the TLR4/MD‐2 receptor complex on the cell surface. This interaction initiates the MyD88‐dependent signalling pathway, resulting in NF‐κB activation and the production of pro‐inflammatory cytokines, including TNF‐α and IL‐5 (Figure 2C).^48^, ^63^, ^64^, ^65^ A parallel TRIF‐dependent pathway may also be activated, but MyD88 is essential for the rapid inflammatory response.^66^, ^67^ Flagellin, the primary component of bacterial flagella, is recognised as a PAMP through two distinct pathways.^68^, ^69^, ^70^ On the one hand, flagellin binds to Toll‐like receptor 5 (TLR5) via a conserved D1 domain containing the QRVRELAV sequence. This interaction recruits the adaptor MyD88, activating NF‐κB and MAPK pathways to induce pro‐inflammatory cytokines such as IL‐8 and IL‐12. On the other hand, intracellular flagellin is detected by neuronal apoptosis inhibitory protein 5 (NAIP5), which oligomerises with NLR damage associated molecular patterns(Nod‐like receptor) family CARD domain‐containing protein 4 (NLRC4) to form the inflammasome. This complex activates Caspase‐1, leading to proteolytic maturation of IL‐1β and IL‐18.^71^, ^72^ The dual recognition of flagellin by TLR5 (extracellular) and NAIP5/NLRC4 (intracellular) enables coordinated innate immune responses. TLR5 signalling primes local inflammation, while NAIP5/NLRC4‐dependent IL‐18 release enhances systemic anti‐tumour immunity by promoting NK cell cytotoxicity and CD8+ T‐cell effector functions.

In addition to TLRs, bacterial components such as peptidoglycan are recognised by cytosolic receptors NOD1/NOD2 (Figure 2D),^49^ which recruit receptor‐interacting protein kinase 2 (RIP2/RICK),^73^ activating NF‐κB and MAPK pathways to induce chemokines (e.g., CXCL1) and antimicrobial peptides like β‐defensins.^74^, ^75^ This signalling cascade drives the expression of chemokines (e.g., CXCL1) and antimicrobial peptides. The interaction between bacteria and host PRRs also plays a critical role in promoting the maturation of DCs. For example, LPS upregulates the surface expression of CD80, CD86 and MHC‐II molecules on DCs through the TLR4‐MyD88 pathway, enhancing their antigen‐presenting capacity.^76^, ^77^, ^78^ Whole bacteria further stimulate DCs to secrete IL‐12p70, which promotes Th1 polarisation and CD8+ T‐cell cytotoxicity.^79^, ^80^ Preclinical studies have shown that natural strains such as *S*. Typhimurium recruit M1‐type macrophages and neutrophils in the TME through TLR4/5 signalling, while reducing the proportion of Tregs, thereby reshaping the immunosuppressive microenvironment.^81^, ^82^, ^83^ Chang et al. revealed that IL‐10R expression on tumour‐infiltrating immune cells exhibits a hysteretic response to intratumour IL‐10 concentration, and an engineered *Salmonella enterica* strain leveraging this property can reconcile the dual challenge in bacterial cancer therapy (BCT) by evading antimicrobial immune defences while promoting anti‐tumour activity, with the IL‐10Rhi state of tumour‐infiltrating immune cells being a ubiquitous trait across most human solid tumour types.^84^

### 2.4 Multifunctional regulation: bacteria as ‘versatile players’

Bacteria not only exert their anti‐tumour effects through targeting and immunomodulation but also initiate multi‐layered attacks on tumours via mechanisms such as toxin secretion and metabolic regulation, resulting in synergistic anti‐tumour effects.^85^ Toxin secretion serves as a critical approach for bacteria to directly kill tumour cells. For instance, *Staphylococcus aureus* α‐haemolysin is identified as a protein drug secreted by anticancer bacteria, which rapidly kills cancer cells, whereas ClyA is endogenously expressed by several *Escherichia* and *Salmonella* strains and induces cellular lysis of infected cells.^86^ Additionally, *Lm* triggers a ROS burst through activation of NADP(+) oxidase and increased intracellular Ca(2+) levels,^87^, ^88^ while *Pseudomonas aeruginosa*’s Azurin protein stabilizes p53, leads to increased expression of Bax and induces cancer cell apoptosis.^89^, ^90^, ^91^ Through their intrinsic biological mechanisms—such as toxin secretion, metabolic interference and immune modulation—bacteria orchestrate a multi‐pronged anti‐tumour response, highlighting their translational promise in cancer therapy. The most well‐known chassis bacterium is *Escherichia coli Nissle 1917* (EcN). EcN is an engineered strain of *Escherichia coli* (*E. coli*) originally isolated by Dr. Alfred Nissle.^92^ It has the characteristic ability to inhibit the in vitro growth of pathogenic *E. coli* and other related species. As a facultative anaerobe, EcN can grow in the hypoxic environments within tumours, utilising these conditions to specifically colonise tumour tissues. Due to its inherent antimicrobial and anti‐inflammatory properties, EcN has been used in the treatment of inflammatory bowel diseases (IBDs).^93^ Subsequent studies have revealed that engineered EcN can express anti‐tumour proteins, such as the azurin protein, which directly inhibits tumour growth.^94^ Additionally, it can express prodrug‐converting enzymes, converting inactive prodrugs into cytotoxic compounds, thereby generating localised therapeutic effects within the tumour. Owing to its potent anti‐tumour effects, EcN is an excellent candidate as a chassis strain for anti‐tumour genetic engineering.^95^

## 3 ENGINEERING STRATEGIES FOR ENHANCING BACTERIAL THERAPY

**FIGURE 3:**
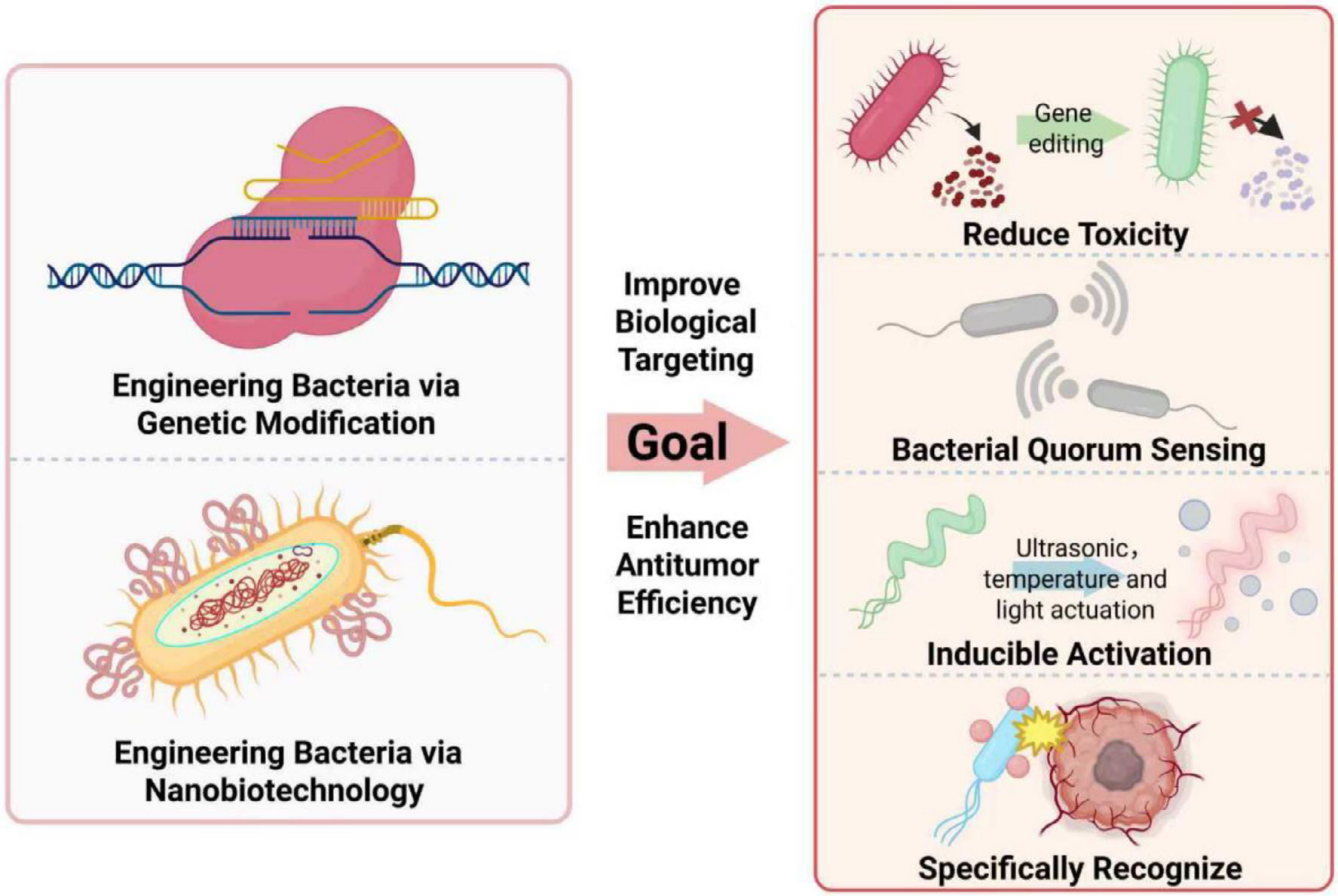
Strategies and objectives for engineering bacteria. Bacteria can be engineered through genetic modification, nanomaterial‐based functionalisation or a combination of these approaches. The primary objectives include (1) reducing bacterial toxicity via gene knockout or modification, (2) enhancing controllability by regulating quorum sensing systems, (3) achieving tuneable bacterial behaviour through diverse engineering strategies and (4) improving tumour cell recognition and targeting efficiency in the tumour microenvironment. Created with https://www.biorender.com.

Bacteria‐based drug delivery systems have gained significant attention due to their unique ability to target specific tissues, particularly tumours. Bacteria, as living organisms, possess inherent properties that can be harnessed for the delivery of therapeutic agents to disease sites, offering distinct advantages over conventional drug delivery systems. These include enhanced tumour targeting, the ability to penetrate solid tumours and the capacity for sustained drug release. Wild‐type bacteria have limited functionality, whereas genetically engineered bacteria demonstrate significant advantages, including reduced toxicity to the host and enhanced anti‐tumour effects. There are two main approaches for modifying engineered bacteria: one is through genetic editing to construct engineered bacteria, and the other is by utilising nanobiotechnology to modify bacteria, thereby enhancing their anti‐tumour capabilities (Figure 3).

### 3.1 Engineering bacteria via genetic modification

Specific genetic editing can reduce the toxicity of bacteria to the host organism. For instance, *S*. Typhimurium typically has certain toxicity to the host, but through genetic engineering, attenuated strains can be developed to enhance their immune effects. *S. Typhimurium* lacking the *aroA* gene showed increased virulence in mouse models, and this strain was able to induce higher levels of the pro‐inflammatory cytokine TNF‐α. Through genetic, phenotypic characteristics, transcriptomic and metabolomic analysis of these strains, it was found that *aroA* deletion caused pleiotropic changes in bacterial cell physiology, lipid and amino acid metabolism, and also increased the response to penicillin, complement and sensitivity to phagocytosis. In addition, *aroA* deletion also affected the expression of virulence‐related genes arnT and *ansB*, as well as pilus phase variation. *S*. Typhimurium with *aroA* gene deletion has important potential applications in vaccines and cancer treatment, especially in bacterial‐mediated tumour treatment.^96^ The phosphotransferase system is a critical glucose uptake system in bacteria, playing an essential role in their survival and replication within host cells. By introducing mutations in the *ptsI* and *crr* genes in *S*. Typhimurium, researchers developed an attenuated strain that exhibited high immunogenicity and protective effects in experimental mice. This strain effectively induced mucosal immune responses and may also be a candidate for a live delivery vector for heterologous antigens.^97^

Certain genetic modifications can restrict bacterial growth in the bloodstream or normal tissues, enhancing their colonisation within the TME. Researchers have engineered *S*. Typhimurium to develop novel anti‐tumour therapeutic approaches. For instance, through genetic modification, a strain of *Salmonella* known as *S*. Typhimurium was created, which is an ‘obligate anaerobe’ capable of growth only under hypoxic conditions. This strain cannot proliferate in the oxygen‐rich environment of normal tissues but survives and thrives in the hypoxic regions of tumours, effectively suppressing tumour growth. Experimental results showed that the YB1 strain significantly inhibited tumour progression in a mouse breast cancer model while leaving normal tissues unharmed.^98^

**FIGURE 4:**
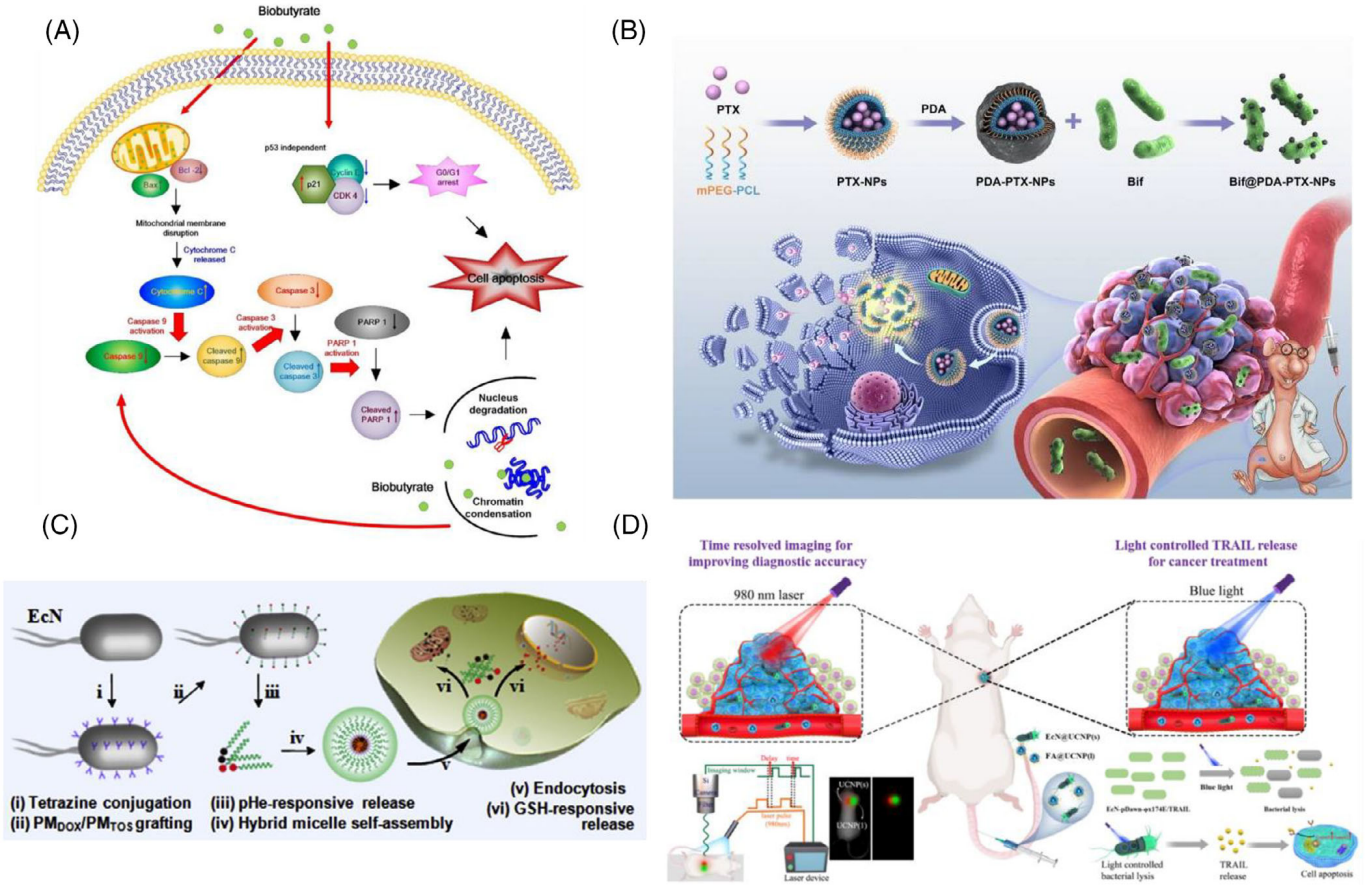
Molecular mechanisms of engineering strategies for enhancing bacterial therapy. (A) Schematic diagram showing the effect of biobutyrate on the mitochondrial apoptosis pathway and the cell cycle regulation. Adapted from ref., 101 with permission of Springer Nature, copyright (2021). (B) Schematic diagram shows the construction of the Bif@PDA‐PTX‐NPs biohybrid and its intelligent responsibility to reductive tumour microenvironment through self‐driven targeting to hypoxic regions of tumour. Adapted from ref., 102 with permission of Dove Medical Press Limited, copyright (2023). (C) Schematic drawing of the bacterial microbots to load, deliver and release active drugs for cancer therapy. Tetrazine derivatives are conjugated onto EcN (i), followed by grafting of norbornene‐terminated PMDOX and PMTOS copolymers by bio‐orthogonal reactions (ii). The PMDOX and PMTOS copolymers are released from the bacterial microbots in response to tumour pHe (iii), followed by self‐assembly into MD/T hybrid micelles (iv). After endocytosis into tumour cells (v), micelles release DOX and TOS in response to cytosolic glutathione peroxidase (GSH) for the potential action of the drugs on the nucleus and mitochondria, respectively (vi). Adapted from ref., 103 with permission of Elsevier, copyright (2018). (D) Light‐controlled engineered bacteria (EcN‐pDawn‐φx174E/TRAIL) were constructed and lysed inducing by a blue light laser, thus achieving the release of tumour necrosis‐related apoptosis‐inducing ligand (TRAIL) for cancer therapy. The released TRAIL could regulate the apoptosis‐related protein expression, thus inducing cancer cell death. Adapted from ref., 104 with permission of American Chemical Society (ACS), copyright (2022).

Probiotics engineered through synthetic biology offer a promising approach to remodelling the TME. The availability of intratumoural L‐arginine is a critical determinant of effective anti‐tumour T‐cell responses. Therefore, increasing the typically low levels of L‐arginine within tumours could significantly enhance the anti‐tumour efficacy of immune checkpoint inhibitors, such as PD‐L1 blocking antibodies.^99^ Using synthetic biology approaches, an engineered probiotic EcN strain was developed, capable of colonising the TME and persistently converting metabolic waste products, such as ammonia, into L‐arginine. This bacterial colonisation within tumours elevated intratumoural L‐arginine levels, enhanced the infiltration of T‐cells into the tumour, and demonstrated significant synergistic effects with PD‐L1 blocking antibodies in tumour eradication.^100^ Through metabolic pathway engineering, EcN was modified to synthesise butyrate (biobutyrate), a compound with potent anti‐cancer properties. Butyrate enhances anti‐tumour therapy by directly promoting CD8+ T‐cell immune responses through the IL‐12 signalling pathway. Under hypoxic conditions, the modified strain (EcN‐BUT) predominantly produced butyrate. When butyrate produced by EcN‐BUT was introduced to human colorectal cancer cells, it caused cell cycle arrest in the G1 phase and induced a p53‐independent mitochondrial apoptotic pathway. In tumour‐bearing mice, administration of EcN‐BUT enabled specific colonisation within tumours and led to a significant reduction in tumour volume by up to 70%. These findings indicate that butyrate‐based BCT opens new avenues for cancer treatment (Figure 4A).^101^

*Vibrio* species possess complex cell‐to‐cell communication systems, such as quorum sensing (QS), which can be harnessed to engineer bacterial populations for coordinated drug delivery and therapeutic protein expression.^105^ A study integrated the *P_BAD_* promoter into the LuxI/LuxR system of *Vibrio* to construct engineered *Salmonella* and tested the functionality of this system within three‐dimensional tumour tissue chips. Results demonstrated that bacteria equipped with communication abilities produced 350 times more protein under optimal induction signals compared to non‐communicating bacteria. Using this cell communication system, bacterial fluorescence expression was enhanced by 40‐fold, and sensitivity to induction molecules increased by over 10 000‐fold. Bacterial communication optimises triggering strategies by enabling activated bacteria to induce entire populations, thereby reducing triggering agent‐induced side effects and offering new insights for the development of novel engineered bacterial strains for cancer therapy.^106^

### 3.2 Engineering bacteria via nanobiotechnology

Currently, the application of nanodrugs is limited due to their instability in delivery.^107^ However, by chemically modifying probiotics, it is possible to create fully active nanodrugs that are more stable and can be specifically targeted to tumours, thus overcoming this limitation and offering significant advantages. For instance, Nguyen et al. combined paclitaxel (PTX)‐loaded liposomal microcargo with tumour‐targeting *S*. Typhimurium. By integrating biotin molecules on bacterial outer membrane proteins with streptavidin‐coated drug‐loaded liposomes, tumour‐therapeutic liposomal bacteria were constructed, enhancing drug migration speed and exhibiting superior tumour‐killing effects compared to conventional liposomal drugs.^108^

The intrinsic properties of nanodrugs allow for TME‐responsive release, thereby minimising drug release in normal tissues. Shi et al. modified *Bifidobacterium infantis* (*Bif*) by synthesising PTX‐loaded nanoparticles (PTX‐NPs) using a methoxy poly (ethylene glycol)‐block‐poly(caprolactone) (mPEG‐PCL) copolymer as the carrier, with a biocompatible polydopamine (PDA) coating. This coating enhanced the adhesion of NPs to bacterial cells and prevented premature drug release. By incubating PDA‐PTX‐NPs with Bif cells, Bif@PDA‐PTX‐NPs hybrids were created. Upon reaching the hypoxic regions of tumour tissues, PTX‐NPs responded to the reductive TME, releasing the drug for tumour cell uptake (Figure 4B).^102^ Similarly, Xie et al. used EcN as a bacterial carrier to deliver doxorubicin (DOX) using acid‐labile linkers, such as maleic anhydride, to create EcN‐ca‐Dox. This approach achieved targeted bacterial accumulation and pH‐induced drug release in the TME, resulting in improved anti‐tumour efficacy in terms of tumour growth inhibition, extended survival and tumour cell apoptosis. Furthermore, antibacterial treatments ensured the clearance of EcN from tumours and other tissues after therapy.^109^ In subsequent studies, Xie et al. optimised the linker and continued using EcN as a bacterial carrier. They immobilised amphiphilic copolymers on the bacterial surface using the acid‐sensitive 2‐propionic‐3‐methylmaleic anhydride (CDM) linker. DOX and α‐tocopheryl succinate were conjugated to PEG via disulphide bonds to form amphiphilic prodrug polymers (PMDOX and PMTOS). Tetrazine and norbornene groups were introduced into EcN and the PMTOS/PMDOX polymers, respectively, allowing site‐specific bio‐orthogonal reactions to create conjugates that retained EcN's activity, motility and tumour‐accumulation capabilities. The PMTOS/PMDOX polymers were released in response to the mildly acidic TME, forming mixed micelles (MD/T) at the tumour site. These micelles enabled intracellular drug release, effectively killing tumour cells (Figure 4C).^103^

Some bacteria‐based anti‐tumour therapies are combined with emerging photothermal materials. Wu et al. designed microrobots by first modifying DOX with maleic anhydride and hydrazine derivatives (Hyd‐SH) using pH‐sensitive amide and imine bonds. The functionalised CA‐Dox‐Hyd‐SH was further coordinated with a photosensitiser, gold nanorods (AuNRs), and then conjugated to the surface of EcN to produce EcN‐Dox‐Au microrobots. Under near‐infrared (NIR) laser irradiation, these microrobots demonstrated enhanced tumour accumulation and penetration into hypoxic tumour cores. After 21 days of treatment with the EcN‐Dox‐Au formulation, complete tumour regression was achieved, with no recurrence observed for at least 53 days.^110^

### 3.3 Integration of nanobiotechnology and genetic modification

The synergistic application of nanobiotechnology and genetic engineering can construct engineered bacteria with more complex functions. Engineering bacteria for visualised and precise drug delivery represents an emerging research frontier. An ideal drug delivery strategy requires the precise and controllable delivery of therapeutic agents to targeted sites.^111^ However, conventional drug delivery systems often lack visualisation capabilities. Recent studies demonstrate that engineered bacteria can serve as drug delivery carriers, enabling precise drug targeting and real‐time visualisation of therapeutic effects through bioimaging techniques. This enables effective monitoring and optimisation of therapeutic outcomes, providing new perspectives and tools for the field of drug delivery, ultimately making treatments more precise and controllable. Zhang et al. developed a light‐controlled engineered bacterial system based on upconversion nanoparticles (UCNPs) for tumour imaging and therapy. Tumour‐targeting molecules (e.g., folic acid) or anaerobic bacteria (EcN) were used to modify UCNPs with distinct luminescence lifetimes for tumour co‐localisation, greatly enhancing the diagnostic precision of time‐resolved imaging. Subsequently, blue light induced the lysis of engineered bacteria (EcN‐pDawn‐φx174E/TRAIL), releasing tumour necrosis‐related apoptosis‐inducing ligand (TRAIL) to trigger tumour cell death. Both in vitro and in vivo results showed that the light‐controlled system achieved a tumour inhibition rate of up to 53%, demonstrating significant therapeutic advantages (Figure 4D).^104^

In order to reduce the issues related to the poor penetration of blue light into human tissues and its potential phototoxicity, Yanhong Zhu and his team developed an engineered bacteria–nanoparticle composite system (EcN_flaB_–UCNPs) based on NIR light control. The engineered EcN carries a blue‐light‐responsive optogenetic element (EL222 protein) and the *flaB* gene. After the complex accumulates in the TME, an external 808 nm NIR light, which has strong penetration, is applied. UCNPs convert the NIR light into blue light, thereby activating EcN expression and the secretion of the immune‐stimulatory factor FlaB. The secreted FlaB targets and binds to TLR5 on the surface of tumour‐associated macrophages (TAMs), triggering an innate immune response via the MyD88‐dependent signalling pathway. This response promotes macrophage polarisation towards the pro‐inflammatory M1 phenotype, releasing cytokines such as IL‐1β, IL‐6 and TNF‐α. Additionally, it activates dendritic cell maturation and recruits cytotoxic CD8+ T‐cells to infiltrate the TME, ultimately synergistically inducing tumour cell death. This system achieves targeted therapy through a spatiotemporally precise light control mechanism, and no significant toxic effects were observed in blood biochemical markers or major organ pathological analyses, confirming its good biosafety.^112^

## 4 CLINICAL APPLICATIONS AND CHALLENGES OF BACTERIA‐BASED TUMOUR THERAPIES

**FIGURE 5:**
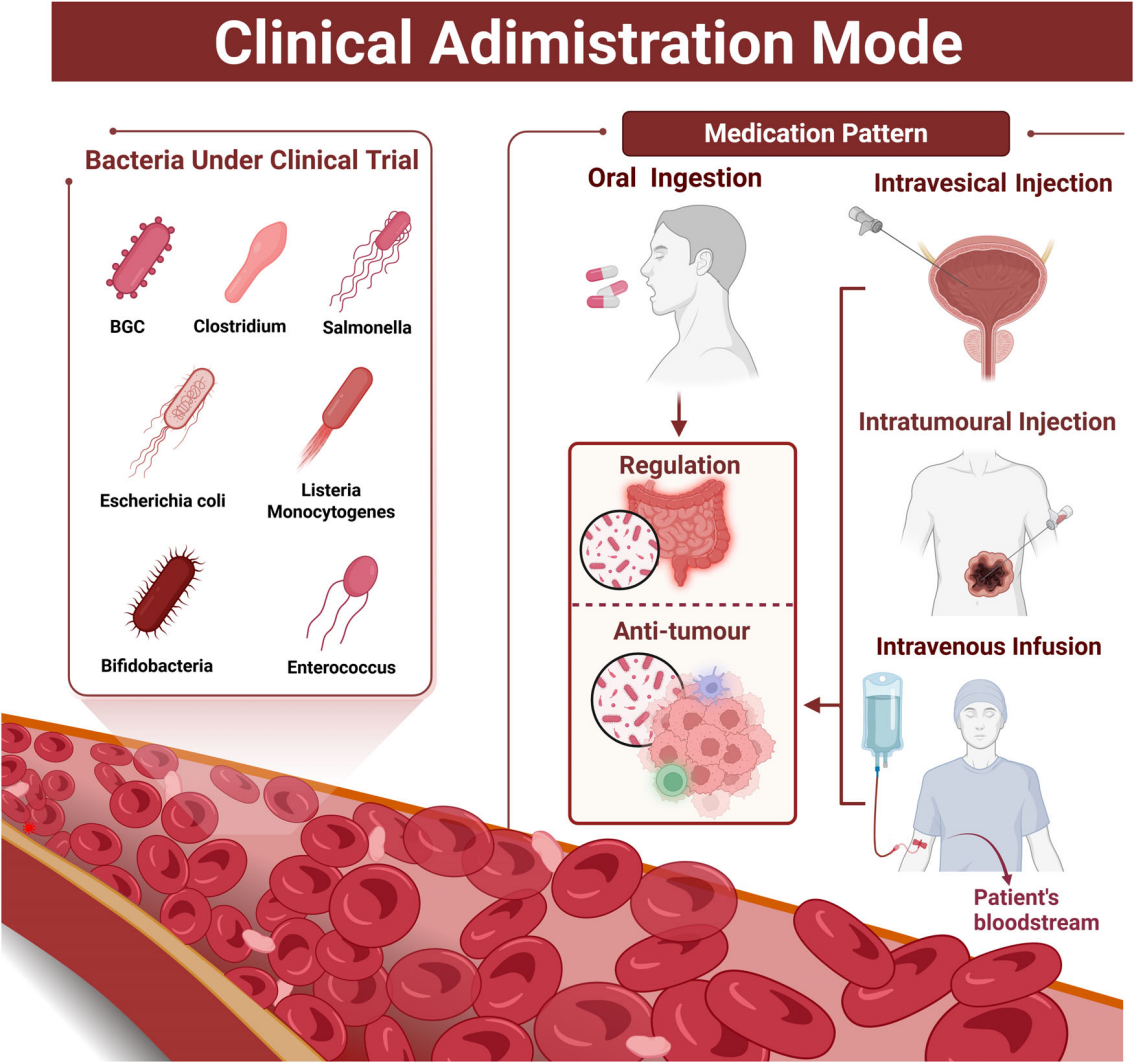
The bacteria‐based cancer therapies that have entered clinical trials, in addition to the already approved Bacillus Calmette–Guérin (BCG), include a wide range of bacterial strains such as *Clostridium*, *Salmonella*, *Listeria*, *Escherichia coli*, *Bifidobacterium* and *Enterococcus*. Currently, the primary administration methods are oral administration, intratumoural injection and intravenous infusion. Oral ingestion primarily utilises the probiotic properties of bacteria to modulate the host's immune system, thereby enhancing the anti‐tumour effects. In contrast, intratumoural and intravenous bacterial injections aim to activate the immune system or directly inflict damage on the tumour, achieving anti‐tumour effects. Created with https://www.biorender.com.

Several bacteria‐based cancer therapies have already entered clinical trial stages, demonstrating promising clinical outcomes. Most of these bacteria have undergone attenuation to minimise side effects on patients. The bacteria currently in clinical trials include *Mycobacterium bovis*, *Clostridium*, *S*. Typhimurium, *Lm*, *E. coli*, *Bifidobacterium* and *Enterococcus* (Table 1). Some of these bacteria have been genetically engineered to enhance their anti‐tumour effects (Figure 5).

**TABLE 1:** Clinical trials of bacteria‐based tumour therapies.

Therapy name	Bacterial strain/components	Indication	Clinical phase/status	Administration routes of bacteria‐based pharmaceuticals	Information source
BCG	Attenuated *Mycobacterium bovis* BCG	High‐risk non–muscle‐invasive bladder cancer (NMIBC)	FDA‐approved since the 1970s (specifically approved in 1976/1977)	Intravesical instillation	^7^, ^113^
*Clostridium novyi* non‐toxin (C. novyi*‐NT*)	C. novyi*‐NT* spores	Refractory solid tumours	Phase I (NCT01924689)	Direct injection into the tumour necrosis area	https://clinicaltrials.gov/study/NCT01924689
		Refractory solid tumours	Phase I (NCT01118819)	Intravenous infusion	https://clinicaltrials.gov/study/NCT01118819
		Solid tumours resistant to standard therapies	Phase I (NCT00358397)	Intravenous infusion	https://clinicaltrials.gov/study/NCT00358397
	C. novyi*‐NT* and pembrolizumab	Advanced solid malignant tumours	Phase I (NCT03435952)	Intratumoural injection	https://clinicaltrials.gov/study/NCT03435952
*Clostridium butyricum* (*C. butyricum*)	The combination of Gemcitabine and teriberilumab with or without *C. butyricum*	Bladder cancer	Phase I (NCT06696742)	Oral ingestion	https://clinicaltrials.gov/study/NCT06696742
	Induction chemotherapy (paclitaxel, cisplatin, 5‐fluorouracil (5‐FU)) and immunotherapy (Sintilimab) with CBM588	Locally advanced oesophageal squamous cell carcinoma	Phase II (NCT06401447)	Oral ingestion	https://clinicaltrials.gov/study/NCT06401447
	Nivolumab combined with ipilimumab, with or without CBM588	Kidney cancer that is stage IV or has spread to other places in the body (advanced)	Phase I (NCT03829111)	Oral ingestion	https://clinicaltrials.gov/study/NCT03829111
	Chemotherapy or chemotherapy combined with anti‐PD‐L1 monoclonal antibody, or chemotherapy combined with anti‐PD‐L1 monoclonal antibody and CBM588	Locally advanced colorectal cancer	Phase II (NCT05914389)	Oral ingestion	https://clinicaltrials.gov/study/NCT05914389
*Clostridium histolyticum* (*C. histolyticum*)	XIAFLEX: Collagenase *C. histolyticum*	Lipoma	Phase II (NCT02249052)	Intratumoural injection	https://clinicaltrials.gov/study/NCT02249052
		Lipoma	Phase II (NCT01613313)	–	https://clinicaltrials.gov/study/NCT01613313
	Collagenase *C. histolyticum*	Uterine leiomyoma (fibroid)	Phase I (‐)	Intratumoural injection	^114^
SYNB1891	EcN engineered with the *dacA* gene, capable of converting ATP into c‐di‐AMP to activate STING, combined with atezolizumab therapy	Metastatic solid neoplasm and lymphoma	Phase I (NCT04167137)	Intratumoural injection	https://clinicaltrials.gov/show/NCT04167137
TAPET‐CD	*Salmonella* Typhimurium (*S*. Typhimurium) with chromosomal deletions of the *purI* and *msbB* genes, resulting in attenuation, and carrying the cytidine deaminase (CD) gene	Malignant solid tumours, melanoma	Phase I (‐)	Intratumoural Injection	^115^
VNP20009	*S*. Typhimurium with chromosomal deletions of *purI* and *msbB* genes resulting in attenuation	Metastatic melanoma, metastatic renal cell carcinoma	Phase I (‐)	Intravenous infusion	^116^
		Metastatic melanoma	Phase I (‐)	Intravenous infusion	^117^
		Advanced solid tumours	Phase I (NCT00006254)	–	https://clinicaltrials.gov/study/NCT00006254
		Advanced or metastatic cancer	Phase I (NCT00004216)	Intravenous infusion	https://cdek.pharmacy.purdue.edu/trial/NCT00004216
		Advanced or metastatic cancer	Phase I (NCT00004988)	Intravenous infusion	https://clinicaltrials.gov/study/NCT00004988
Saltikva	Saltikva: Attenuated *S*. Typhimurium containing the human IL‐2 gene	Metastatic gastrointestinal cancer	Phase I (‐)	Oral ingestion	^118^
		Metastatic pancreatic cancer	Phase II (NCT04589234)	Oral ingestion	https://clinicaltrials.gov/study/NCT04589234
				Oral	^119^
CVD908ssb‐TXSVN	CVD908ssb‐TXSVN: Survivin‐expressing attenuated S. Typhimurium	Multiple myeloma	Phase I (NCT03762291)	Oral ingestion	https://clinicaltrials.gov/study/NCT03762291
VXM01	VXM01: *S*. Typhimurium Ty21a carrying an expression plasmid encoding VEGFR2	Advanced pancreatic cancer	Phase I (NCT01486329)	Oral ingestion	^119^
		Metastatic colorectal cancer with liver metastasis	Phase I (NCT02718430)	Oral ingestion	https://clinicaltrials.gov/study/NCT02718430
		Glioblastoma	Phase I (NCT02718443)	Oral ingestion	https://clinicaltrials.gov/study/NCT02718443
	VXM01 in combination with anti‐PDL1 avelumab treatment	Glioblastoma	Phase I/II (NCT03750071)	Oral ingestion	^120^
ADXS11‐001 (Lovaxin C)	Attenuated *Listeria monocytogenes* (*Lm*) expressing a fusion of the HPV 16 E7 antigen and a truncated Listeriolysin O (tLLO) protein	Cervical cancer	Phase III (NCT02853604)	Intravenous infusion	^121^, ^122^
		Unresolved or recurrent (relapsed) cervical cancer	Phase II (NCT01266460)	Intravenous infusion	https://clinicaltrials.gov/study/NCT01266460
		Oropharyngeal squamous cell carcinoma	Phase II (NCT02002182)	–	https://clinicaltrials.gov/study/NCT01598792
		HPV‐positive oropharyngeal squamous cell carcinoma	Phase II (NCT02002182)	Intravenous infusion	https://clinicaltrials.gov/study/NCT02002182
		Previously treated, unresectable, persistent/recurrent loco‐regional or metastatic anal cancer	Phase II (NCT02399813)	Intravenous infusion	^123^
		Persistent, metastatic or recurrent squamous cell carcinoma and non‐squamous cell carcinoma, adenosquamous carcinoma or cervical adenocarcinoma	Phase I (NCT02164461)	Intravenous infusion	https://clinicaltrials.gov/study/NCT02164461
		Recurrent/persistent or metastatic cervical squamous cell carcinoma or metastatic human papillomavirus (HPV) and head and neck squamous cell carcinoma	Phase I/II (NCT02291055)	Intravenous infusion	https://clinicaltrials.gov/study/NCT02291055
		Cervical intraepithelial neoplasia (CIN) 2+	Phase II (NCT01116245)	Intravenous infusion	https://clinicaltrials.gov/study/NCT01116245
	Combination therapy of ADXS11‐001 with mitomycin, 5‐FU and intensity‐modulated radiation therapy	Anal cancer	Phase I/II (NCT01671488)	Intravenous infusion	https://clinicaltrials.gov/study/NCT01671488
	ADXS11‐001 in combination with Pemetrexed	Human papillomavirus positive, non‐squamous, non–small‐cell lung cancer	Phase II (NCT02531854)	–	https://clinicaltrials.gov/study/NCT02531854
CRS‐207	CRS‐207 (attenuated Lm releasing mesothelin)	Advanced ovarian cancer or pancreatic cancer, non–small‐cell lung cancer, or advanced malignant epithelioid mesothelioma	Phase I (NCT00585845)	Intravenous infusion	https://clinicaltrials.gov/study/NCT00585845
	CRS‐207 or CRS‐207 in combination with GVAX pancreatic vaccine with cyclophosphamide (CY) and chemotherapy	Metastatic pancreatic cancer	Phase II (NCT02004262)	Intravenous infusion	https://clinicaltrials.gov/study/NCT02004262
	GVAX pancreatic vaccine (with CY) or CRS‐207 in combination with GVAX pancreatic vaccine (with CY)	Metastatic pancreatic cancer	Phase II (NCT01417000)	Intravenous infusion	^124^
	GVAX pancreatic vaccine (with CY) and CRS‐207 in combination with or without nivolumab	Metastatic pancreatic cancer	Phase II (NCT02243371)	Intravenous infusion	https://clinicaltrials.gov/study/NCT02243371
	Tadalafil or pembrolizumab or ipilimumab or CRS‐207	Metastatic pancreatic cancer	Phase II (NCT05014776)	Intravenous infusion	https://clinicaltrials.gov/study/NCT05014776
	CRS‐207 (with or without CY) in combination with standard chemotherapy (pemetrexed and cisplatin)	Malignant pleural mesothelioma in adults	Phase I (NCT01675765)	Intravenous infusion	https://clinicaltrials.gov/study/NCT01675765
	CRS‐207 with or without Nivolumab, ipilimumab and CY/GVAX pancreatic vaccine	Metastatic pancreatic cancer	Phase II (NCT03190265)	Intravenous infusion	https://clinicaltrials.gov/study/NCT03190265
	Epacadostat, pembrolizumab and CRS‐207, with or without CY/GVAX pancreatic vaccine	Metastatic pancreatic cancer	Phase II (NCT03006302)	Intravenous infusion	https://clinicaltrials.gov/study/NCT03006302
	CRS‐207 in combination with pembrolizumab	Malignant pleural mesothelioma	Phase II (NCT03175172)	Intravenous infusion	https://clinicaltrials.gov/study/NCT03175172
	CRS‐207 in combination with pembrolizumab	Recurrent or metastatic gastric cancer, gastroesophageal junction cancer or oesophageal cancer	Phase II (NCT03122548)	Intravenous infusion	https://clinicaltrials.gov/study/NCT03122548
	CRS‐207 in combination with epacadostat	Platinum‐resistant ovarian cancer, fallopian tube cancer or peritoneal cancer	Phase I/II (NCT02575807)	Intravenous infusion	https://clinicaltrials.gov/study/NCT02575807
CRS‐100 (ANZ‐100)	CRS‐100: Live attenuated *Lm* with deletion of virulence genes *actA* and *internalin B*	Treatment‐refractory cancer and liver metastasis	Phase I (NCT00327652)	Intravenous infusion	[^125^]
JNJ‐809(JNJ‐64041809)	JNJ‐809: live‐attenuated double deletion monocytic proliferation Lm	Metastatic castration‐resistant prostate cancer	Phase I (NCT02625857)	Intravenous infusion	https://clinicaltrials.gov/study/NCT02625857
	Apalutamide in combination with or without JNJ‐809	Metastatic castration‐resistant prostate cancer	Phase II (NCT02906605)	–	https://clinicaltrials.gov/study/NCT02906605
ADXS‐NEO	Lm expressing tumour‐associated antigens, with or without pembrolizumab	Advanced or metastatic solid tumours	Phase I (NCT03265080)	Intravenous infusion	https://clinicaltrials.gov/study/NCT03265080
APS001F	APS001F: *Bifidobacterium longum* genetically engineered to express cytosine deaminase that converts 5‐FC to 5‐fluorouracil	Advanced and/or metastatic solid tumours	Phase I/II (NCT01562626)	–	https://clinicaltrials.gov/study/NCT01562626
bacTRL‐IL‐12	bacTRL‐IL‐12: *Bifidobacterium longum* used to deliver plasmid DNA encoding the IL‐12 transgene	Advanced refractory solid tumours	Phase I (NCT04025307)	Intravenous infusion	https://clinicaltrials.gov/study/NCT04025307
MRx0518	MRx0518: *Enterococcus gallinarum*	Solid tumours	Phase I (NCT03934827)	Oral ingestion	https://clinicaltrials.gov/study/NCT03934827
	MRx0518 combined with large fractionated neoadjuvant radiotherapy	Resectable pancreatic cancer	Phase I (NCT04193904)	Oral ingestion	https://clinicaltrials.gov/study/NCT04193904
	MRx0518 combined with Avelumab	Metastatic urothelial carcinoma	Phase II (NCT05107427)	Oral ingestion	https://clinicaltrials.gov/study/NCT05107427
	MRx0518 combined with Pembrolizumab	Non–small‐cell lung cancer, renal cell carcinoma, bladder cancer or melanoma	Phase I/II (NCT03637803)	Oral ingestion	https://clinicaltrials.gov/study/NCT03637803

### 4.1 Attenuated *Mycobacterium bovis* Bacillus Calmette–Guérin

A well‐known bacteria‐based cancer therapy is Bacillus Calmette–Guérin (BCG), which is the gold standard for the treatment of high‐risk non–muscle‐invasive bladder cancer (NMIBC).^7^ BCG, as the gold‐standard bacteria‐based immunotherapy for this disease, has been widely used in clinical practice. A prospective randomised study compares single‐course intravesical BCG (induction only) with BCG plus maintenance therapy in high‐risk NMIBC, finding that maintenance BCG significantly reduces recurrence rate (19.2% vs. 55.6%, *p* = .01) and improves 5‐year recurrence‐free survival (78% vs. 41%); however, there is no significant difference in tumour progression rate or 5‐year PFS between the two groups, and maintenance BCG is associated with a significantly higher incidence of local adverse events.^126^ Boban et al. explored urinary microbiota changes during six induction cycles of BCG therapy in NMIBC patients, finding a significant decrease in biodiversity (Shannon index) in 10 out of 12 patients during the first week (*p* = .021), and differences in pre‐therapy microbiota composition between responders and non‐responders, with non‐responders showing 12‐fold higher abundance of the genus *Aureispira* and 27‐fold lower abundance of the species *Negativicoccus succinivorans* (both *p* < .001), suggesting the urinary microbiota's role in BCG therapy outcome.^127^ To further enhance therapeutic efficacy, a recent study employed a biotin–streptavidin strategy to load PLGA‐encapsulated DOX nanoparticles onto the surface of live BCG bacteria (DOX@BCG) via biotinylated *Mycobacterium* polyclonal antibodies as linkers. BCG's natural tropism for bladder epithelium (mediated by fibronectin attachment protein and Ag85 family proteins binding to fibronectin via integrin α5β1) facilitates targeted DOX@BCG delivery to tumour cells while improving intratumoural drug transport. This triple‐action approach synergises BCG‐mediated immunotherapy, DOX chemotherapy and DOX‐induced immunogenic cell death, effectively suppressing tumour progression and extending survival in three orthotopic bladder cancer models: BBN‐induced mouse model, MNU‐induced SD rat model and MB49 cell transplantation mouse model. Additionally, DOX@BCG demonstrates enhanced biosafety (reduced systemic DOX exposure and no obvious damage to major organs), improved treatment tolerance (lower incidence of anaemia and abnormal leukocyte levels compared to BCG alone or BCG+DOX mixture) and establishes robust anti‐tumour immunity within the TME. These findings underscore the clinical potential of live BCG as a versatile intravesical drug delivery system for improving NMIBC therapy.^128^

### 4.2 Clostridium

The species of *Clostridium* that have been used in cancer therapy include attenuated anaerobic bacterium *C. novyi‐NT*, *Clostridium butyricum* (*C. butyricum*) and *Clostridium histolyticum* (*C. histolyticum*). *C. novyi‐NT*, generated by deleting the phage carrying α‐toxin to eliminate pathogenicity, has been shown to precisely germinate in and eradicate treatment‐resistant hypoxic tumours in various experimental animal models (e.g., mice, rabbits, rats) and spontaneously occurring canine sarcomas.^129^ Due to the excellent properties of *C. novyi‐NT*, its spores have been developed for tumour therapy, as they are easier to store, handle and have superior stability under non‐permissive conditions. However, the poor colonisation ability of the spores in small tumours lacking hypoxic/necrotic areas and the low fraction of systemically injected spores reaching tumours remain challenges; additionally, combination bacteriolytic therapy (COBALT) with chemotherapy, radiotherapy or immunotherapy is often required to improve efficacy, and strategies based on this bacterium for tumour therapy still require continuous optimisation.^130^

*C. butyricum* modulates host immunity by producing SCFAs, including butyrate and acetate, and can also regulate gut microbiota composition. Preclinical studies have shown that *C. butyricum* exhibits direct anti‐tumour activity against colon cancer, and its spores encapsulated with prebiotic dextran (spores‐dex) can specifically enrich in colon tumours after oral administration, inhibit tumour growth (up to 89% in subcutaneous models and 65% in orthotopic models), and regulate gut microbiota by augmenting the abundance of SCFA‐producing bacteria (e.g., *Eubacterium*, *Roseburia*). Ongoing clinical studies typically involve oral administration of *C. butyricum* MIYAIRI 588 (CBM588), a live biotherapeutic strain, to enhance the therapeutic efficacy of ICB in cancer patients—especially those receiving proton pump inhibitors (PPIs) or concurrent PPIs and antibiotics, by reducing harmful oral‐related pathobionts in the gut.^131^, ^132^ A randomised phaseⅠ trial showed that CBM588 combined with dual ICB (nivolumab + ipilimumab) significantly prolonged PFS (12.7 vs. 2.5 months, *p* < .001) in metastatic renal cell carcinoma patients. Some research aimed at optimising *C. butyricum*‐based anti‐tumour therapies (e.g., prebiotic encapsulation, combination with ICB) has also demonstrated promising results, highlighting the important role of gut microbiota in anti‐tumour responses.^133^

*C. histolyticum* collagenase (CCH), approved by the FDA in 2013 for the minimally invasive treatment of stable‐phase Peyronie’ s disease (PD) with dorsal/lateral penile curvature >30°, has preclinical potential to degrade tumour stroma (e.g., in pancreatic cancer) by targeting type I and III collagens; however, clinical translation remains limited due to a lack of large‐scale clinical trials and concerns about potential toxicity in TMEs.^134^

### 4.3 Escherichia coli

EcN, a non‐pathogenic probiotic strain with a long history of safe use in IBD and other gastrointestinal disorders,^89^ is now engineered as a versatile platform for cancer immunotherapy.^93^

In addressing the immunosuppressive ‘cold tumour’ microenvironment, SYNB1891, an engineered EcN strain genetically modified to express DacA (a diadenylate cyclase from *Lm*) under the control of the hypoxia‐inducible *P*_fnrS_ promoter, converts ATP into cyclic‐di‐AMP (c‐di‐AMP) specifically in the hypoxic TME to activate the STING pathway in phagocytic APCs.^135^ Additionally, SYNB1891 incorporates dual biocontainment features—deletions of *thyA* (thymidylate synthase) and *dapA* (4‐hydroxy‐tetrahydropicolinate synthase)—to prevent extra‐tumoural proliferation, and all antibiotic resistance genes were removed to meet regulatory standards. In a Phase I trial (NCT04167137), intratumoural SYNB1891 (doses ranging from 1 × 10^6^ to 3 × 10^8^ live cells) as monotherapy or in combination with atezolizumab induced upregulation of interferon‐stimulated genes (ISGs), type I interferon (IFNα/β) signalling, and T‐cell response genes (e.g., CD4, CD8, granzyme B) in tumour biopsies 7 days post‐treatment. Serum cytokines (TNFα, IL‐6, IFNγ, IL‐1RA) also showed dose‐related increases, confirming STING pathway engagement. Notably, no SYNB1891‐related infections were observed, and the bacterium was not detected in blood at 6 or 24 h after injection or in tumour tissue 7 days after the first dose, validating its safety profile. Preliminary data suggested clinical benefit, with 4 patients (13%) achieving stable disease (SD) for more than 2 months—including one patient with small‐cell lung cancer (SCLC) who maintained SD for over 363 days—despite prior resistance to PD‐1/PD‐L1 inhibitors. The combination with atezolizumab was well tolerated, with no dose‐limiting toxicities observed. However, the trial was terminated early due to the sponsor's strategic shift, precluding determination of the maximum tolerated dose.^136^ Beyond direct anti‐tumour applications, the probiotic EcN is clinically investigated (NCT02706184) to mitigate chemotherapy‐induced diarrhoea in gastrointestinal cancer patients receiving 5‐fluorouracil (5‐FU)‐based regimens, leveraging its safety profile and preclinical efficacy in preserving intestinal barrier function.

### 4.4 *Salmonella* Typhimurium

*S*. Typhimurium has been widely applied in tumour therapy, with one well‐known strain being VNP20009. VNP20009 is an attenuated *S*. Typhimurium strain generated by chromosomal deletions of the *purI* and *msbB* genes. The deletion of *purI* renders the bacteria dependent on an external source of adenine, limiting its colonisation to purine‐rich TME. The loss of the *msbB* gene results in the absence of myristoylation of the lipid A component of LPS, thereby reducing the toxicity associated with LPS.^137^, ^138^ VNP20009 has undergone extensive clinical trials, and numerous studies have been conducted based on VNP20009 for the development of novel therapies. TAPET‐CD therapy, based on the attenuated *S*. Typhimurium strain VNP20009, was among the first to validate the feasibility of the ‘bacterial vector‐prodrug conversion’ strategy. The genetic attenuation design of VNP20009, involving the deletion of the *purI* (purine biosynthesis gene, rendering the strain purine‐dependent to limit proliferation) and *msbB* (lipid A biosynthesis gene, reducing endotoxicity) genes, reduced systemic toxicity risks. Notably, TAPET‐CD does not directly express 5‐FU but instead expresses cytidine deaminase (CD).^139^ A Phase I clinical trial of VNP20009 in patients with metastatic melanoma demonstrated favourable tumour colonisation (bacterial counts up to 10^6^ CFU/g tumour tissue) but showed side effects such as fever, chills and nausea (confirmed to be related to bacterial systemic dissemination rather than just colonisation). Despite the lack of significant clinical efficacy (no objective tumour responses), the use of *S*. Typhimurium carrying CD to successfully convert the non‐toxic prodrug 5‐fluorocytosine (5‐FC) into the highly toxic anticancer drug 5‐FU within tumour sites represents a breakthrough in achieving localised and precise tumour targeting.^115^, ^140^

Saltikva is a genetically engineered attenuated *S*. Typhimurium strain capable of expressing human interleukin‐2 (IL‐2). The strain was constructed by transforming the plasmid pYA292, which carries a truncated human IL‐2 gene and the *asd* gene, into the χ4550 strain (attenuated via deletion of *cya* and *crp* genes, which are critical for virulence). The survival of the strain relies on the presence of the *asd* gene (required for cell wall synthesis) on the plasmid, as the χ4550 strain itself is *asd*‐deficient—this design ensures the genetic stability of the engineered bacteria in vivo (plasmid loss leads to bacterial death). A Phase I clinical trial indicated that although patients with metastatic gastrointestinal cancers treated with single‐dose Saltikva did not achieve complete or partial remission, they exhibited statistically significant increases in circulating NK cells and NK‐T‐cells (12.1% vs. 14.6%, *p* = .02; 3.4% vs. 5.6%, *p* = .02), suggesting partial immunotherapeutic potential.^118^

VXM01 is a *S*. Typhimurium strain engineered to carry a eukaryotic expression plasmid encoding VEGFR2, enabling targeted expression of VEGFR2. Preclinical and early exploratory studies suggest it may enhance the generation of multifunctional VEGFR2‐reactive CD8+ and CD4+ T effector cell responses, with preliminary evidence of good safety.^8^

CVD908ssb‐TXSVN is a genetically modified attenuated *Salmonella enterica* serovar Typhi (*S*. Typhi) strain, with the strain engineered to express survivin to trigger an immune response against survivin‐positive tumour antigens. In mouse lymphoma and neuroblastoma models, it induced a robust anticancer effect by activating survivin‐specific immune responses.^141^

### 4.5 Listeria monocytogenes

Clinically, ADXS11‐001 (Lovaxin C), an attenuated *Lm* expressing HPV16 E7 fused to truncated LLO (tLLO), has demonstrated potential in HPV‐associated cancers. It activates dual MHC‐I/II pathways to induce tumour‐specific T‐cells while suppressing Tregs and MDSCs.^142^ Clinical trials have validated its efficacy: In a Phase II trial (NCT01266460) for recurrent/refractory cervical cancer, the 12‐month overall survival (OS) rate was 38% (*n* = 19), with a median OS of 6.1 months and median PFS of 2.8 months.^143^ Additionally, ADXS11‐001 has demonstrated good tolerability, with a safety profile consistent with its application in other HPV‐associated malignancies—most treatment‐related adverse events (TRAEs) were grade 1–2 (chills, pyrexia, nausea), and grade 3 TRAEs occurred in 38% of patients, with only two cases of grade 4 events.^144^ In the Phase II clinical trial for anal cancer (NCT02399813), the median OS was 12.6 months.^145^ From a safety perspective, adverse events associated with the vaccine were predominantly grade 1–2 (e.g., fever, chills, hypotension), with severe toxicities (grade 3–4) occurring in 30.6% of patients (10 grade 3, 1 grade 4) . A Phase I/II trial (NCT02291055) investigating the efficacy and safety of ADXS11‐001 in combination with the PD‐L1 inhibitor durvalumab in patients with cervical cancer or HPV‐positive head and neck cancers is underway.

Another representative therapeutic agent, CRS‐207, achieves a significant reduction in bacterial pathogenicity and intercellular dissemination capacity (virulence reduced by 1000‐fold in mice) through the knockout of two virulence genes, *actA* and *inlB (ΔactA/ΔinlB)*. Concurrently, by expressing human mesothelin, it targets tumour cells with high mesothelin expression, such as those in pancreatic cancer and mesothelioma. Upon infecting APCs, CRS‐207 activates mesothelin‐specific CD4+ and CD8+ T‐cell immune responses, thereby inducing tumour cell killing.^146^ Current research efforts are primarily focused on the development of combination therapies. In the Phase II trial for metastatic pancreatic cancer (NCT01417000), the combination of CRS‐207 with the GVAX vaccine demonstrated a median OS of 6.1 months, which was superior to the median survival of 3.9 months observed with GVAX alone (hazard ratio [HR] .59, *p* = .02).^147^ The combination of CRS‐207 and chemotherapy (pemetrexed + cisplatin) demonstrated significant efficacy in unresectable malignant pleural mesothelioma, with a disease control rate of 86%, median OS of 14.7 months and induction of immune activation in the TME along with objective tumour responses, with no unexpected serious adverse events observed.^148^ Additionally, the combination of CRS‐207 with chemotherapy, radiotherapy or dual immune checkpoint inhibitors is under exploration. For instance, a Phase II trial (NCT02243371) aims to evaluate the safety, immune responses and survival benefits of the GVAX pancreas vaccine (combined with cyclophosphamide) and CRS‐207 (an attenuated *Lm*‐based vaccine) with or without nivolumab in previously treated patients with metastatic pancreatic ductal adenocarcinoma (PDAC). Although some Phase III trials have not met their primary endpoints, *Lm*‐based therapies continue to offer a unique strategy for the treatment of solid tumours, and further optimisation holds promise for driving clinical breakthroughs.

### 4.6 Bifidobacterium

*Bifidobacterium* are commonly found in the human gut microbiota and are considered beneficial probiotics.^107^, ^149^ These bacteria primarily protect cells from oxidative stress by increasing the levels of antioxidant enzymes, such as GSH, superoxide dismutase and catalase.^150^ Additionally, they reduce inflammation by activating anti‐inflammatory cytokines (such as IL‐10) and decreasing the expression of pro‐inflammatory cytokines (such as IL‐6). Studies have shown that *Bifidobacterium* can reduce cancer cell proliferation by inhibiting growth factor signalling and inducing mitochondria‐mediated apoptosis, exhibiting strong anti‐tumour effects.^151^

Currently, a drug derived from *Bifidobacterium*, APS001F, has entered the clinical trial stage. APS001F is a genetically engineered strain of *Bifidobacterium longum *that expresses a mutated cytosine deaminase with an active site mutation to enhance enzyme activity.^152^ This enzyme converts the non‐toxic prodrug 5‐FC into the cytotoxic anticancer drug 5‐FU. APS001F is administered intravenously (i.v.), and after selectively colonising the hypoxic regions of solid tumours, it converts systemically delivered 5‐FC (administered intraperitoneally or orally) into 5‐FU locally within tumour tissues. This targeted production of 5‐FU kills tumour cells while minimising systemic toxicity. APS001F is currently in Phase Ⅰ/ Ⅱ clinical trials in the United States (NCT01562626).

BacTRL‐IL‐12 (a *Bifidobacterium longum‐*based ILinterleukin‐12 delivery system) has entered a Phase I clinical trial (NCT04025307) for advanced refractory solid tumours. This engineered bacterium is administered via intravenous infusion and designed to colonise tumour sites, where it locally expresses IL‐12 to activate anti‐tumour immunity. Preclinical studies support the rationale of using *Bifidobacterium* as a tumour‐targeting vector: its anaerobic nature enables selective accumulation in hypoxic TMEs, and cytokine delivery (e.g., IL‐12) can potentiate immune activation against tumours. The trial aims to evaluate safety, tolerability and preliminary efficacy in patients with metastatic cancers, highlighting the translational potential of bacterial cytokine therapy.

### 4.7 Enterococcus

The gut microbiome is closely associated with the occurrence and development of tumours, and it can regulate the response of solid tumours to immunotherapy. Preclinical studies have shown that MRx0518, a live biotherapeutic product consisting of a single strain of *Enterococcus gallinarum* derived from the gut microbiome, reduces tumour growth by 35%–51% in syngeneic mouse models of various cancers, including breast, renal and lung carcinomas. Its anti‐tumour mechanism involves activating the TLR5/NF‐κB signalling pathway via bacterial flagellin, which increases the infiltration of CD4+ T‐cells, CD8+ T‐cells and NK cells into tumours and elevates the CD8+ T‐cell:Treg ratio (rather than simply reducing Tregs).^153^, ^154^ Additionally, MRx0518 induces pro‐inflammatory cytokines such as IL‐8, TNF‐α and IL‐6, further enhancing anti‐tumour immunity. MRx0518 is currently undergoing Phase I/II clinical research for multiple solid tumours. Key trials include combination therapy with pembrolizumab in patients with immune checkpoint inhibitor (ICI)‐refractory cancers (e.g., renal cell carcinoma, non‐small–cell lung cancer) and neoadjuvant use in resectable pancreatic cancer . Administered orally at doses of 1 × 10^10^ to 1 × 10^11^ CFU twice daily, it has demonstrated a favourable safety profile with only grade 1–2 adverse events and preliminary efficacy signals, including SD for ≥6 months in 4/16 evaluable renal cell carcinoma patients. These findings support its potential as a novel microbiome‐based anti‐cancer therapy.^155^

## 5 BACTERIA‐BASED MULTI‐MODAL SYNERGISTIC TUMOUR THERAPY

**FIGURE 6:**
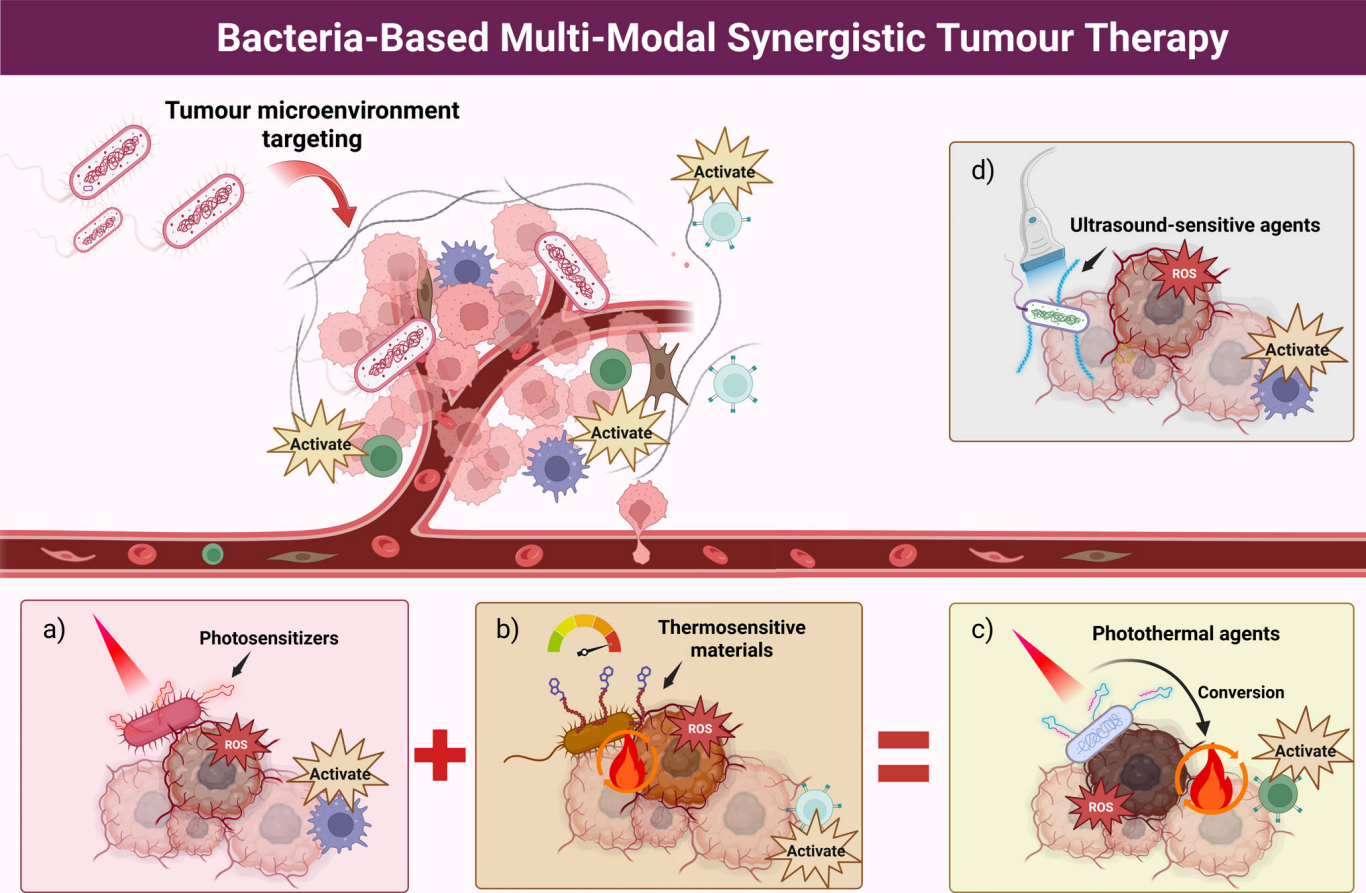
Bacteria can be used for multi‐effect synergistic cancer therapy. Bacteria themselves possess immune activation properties, which can stimulate the body's immune system to achieve tumour cell killing. (A) By using bacteria to deliver photosensitisers, immune and photodynamic therapy can be coordinated under stimulation by specific wavelengths. (B) Bacteria can be modified with thermosensitive materials, enabling immune and thermodynamic synergistic therapy through the control of local tumour temperature. (C) Combining bacteria with photothermal conversion materials allows for multi‐effect synergistic treatment, such as tumour photothermal therapy, maximising the tumour‐killing effect. (D) Bacteria can be utilised for the delivery of sonosensitisers or by constructing vibration‐sensitive bacteria, enabling immune and sonodynamic synergistic therapy under localised ultrasound stimulation. Created with https://www.biorender.com.

Based on the inherent immunity of bacteria, bacterial‐mediated synergistic therapies can be utilised, including PDT, TDT, SDT and immunotherapy. Increasing evidence from research suggests that bacteria can facilitate multi‐modal coordinated treatments for tumours, thereby achieving maximal tumouricidal effects (Figure 6).

### 5.1 Engineered bacteria for combination of PDT and immunotherapy

Engineered bacteria can be utilised for PDT in combination with immunotherapy. PDT mainly relies on the accumulation of photosensitiser drugs in tumour tissue, which, after injection, is significantly higher in tumour tissue than in the surrounding normal tissue. Tumour tissue is then irradiated with a specific wavelength of laser light at an appropriate time to activate the photosensitiser, generating ROS that specifically destroy tumour cells and tumour‐associated blood vessels.^156^, ^157^ Effective targeted delivery of photosensitisers and controlled release of ROS are critical for the success of PDT.

**FIGURE 7:**
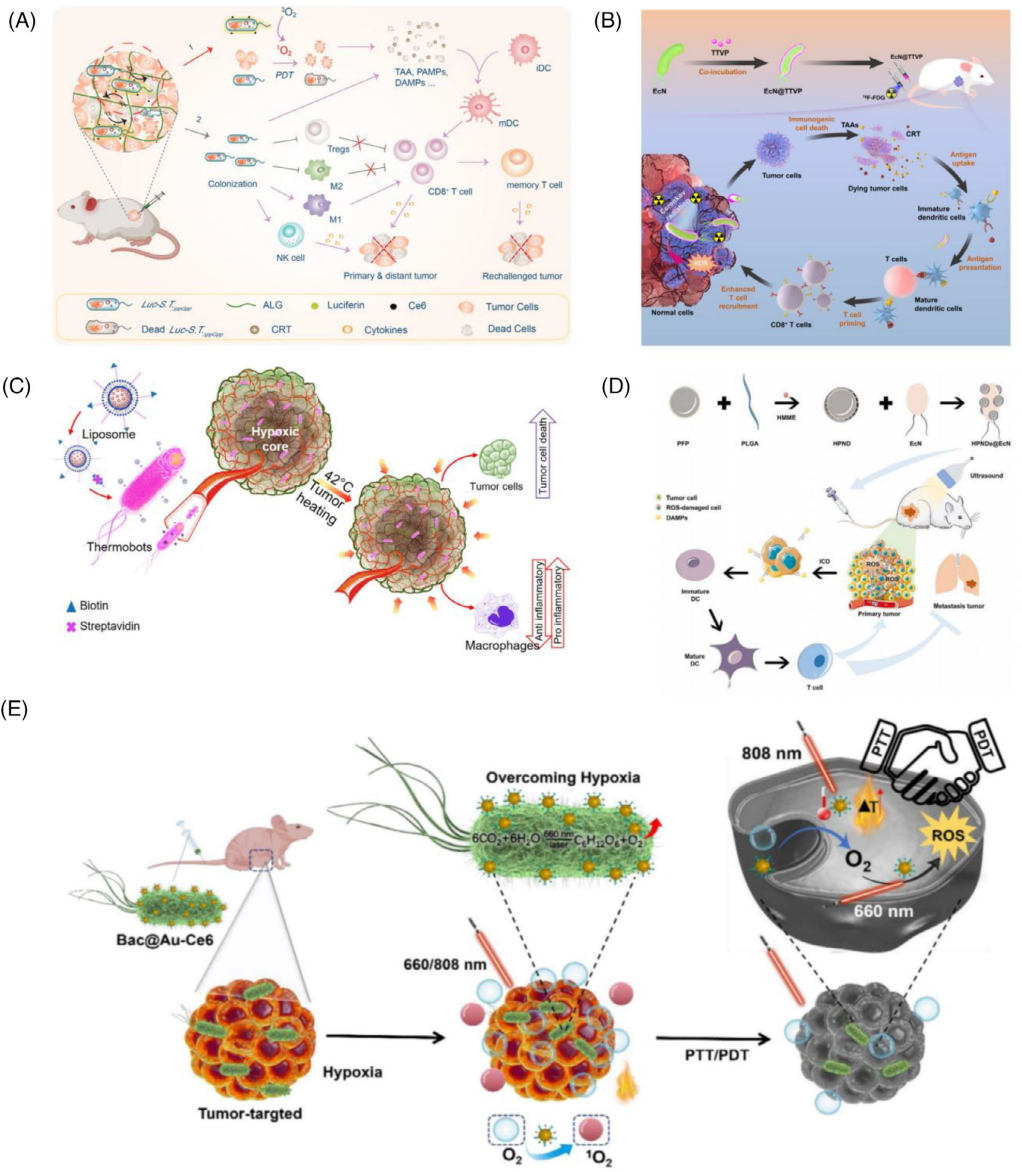
Bacterial‐based multi‐effect synergistic therapy. (A) Schematic diagram showing the effect of Luc‐S.T.ΔppGpp for immune and PDT synergistic therapy in tumours. Adapted from ref., 158 with permission of Elsevier, copyright (2022). (B) Schematic diagram showing the mechanism of EcN@TTVP combined with radioactive drug 18F‐fluorodeoxyglucose for tumour killing at the tumour site. Adapted from ref., 160 with permission of Elsevier, copyright (2023). (C) Schematic diagram showing the mechanism of tumour thermosensitive therapy mediated by thermobots based on S. Typhimurium. Adapted from ref., 161 with permission of Springer Nature, copyright (2018). (D) Schematic diagram showing the mechanism of tumour sonosensitive therapy mediated by HPNDs@EcN. Adapted from ref., 162 with permission of Springer Nature, copyright (2024). (E) Mechanism of PDT and PTT synergistic therapy using Bac@Au‐Ce6. Adapted from ref., 163 with permission of Ivyspring International Publisher, copyright (2023).

Bacteria, due to their strong TME targeting ability and immunoactivation potential, are being explored as adjuncts to PDT for synergistic treatment. Yang et al. created a bioluminescent bacterium by transforming a plasmid expressing firefly luciferase into an attenuated *S*. Typhimurium strain Δ*ppGpp*, resulting in the formation of *Luc*‐*S.T*._Δ_ *_ppGpp_*. *Luc*‐*S.T*._Δ_ *_ppGpp_* in tumours acts as an internal light source in the presence of D‐luciferin, enabling uniform illumination of the tumour, activating chlorin e6 (Ce6) and generating ROS at the tumour site to induce tumour cell death. Additionally, the colonised *Luc*‐*S.T*._Δ_ *_ppGpp_* significantly enhances the immune response, contributing to a synergistic therapeutic effect that inhibits the growth of various tumours, including CT26 tumours and black B16 tumours in mice, as well as large VX2 tumours in rabbits (Figure 7A).^158^ Guo et al. utilised *S*. Typhimurium to express the fluorescently activated protein, which, when combined with fluorogen such as malachite green, generates ROS. The ROS produced by PDT induces the death of cancer cells and the accumulated bacteria. PAMPs and damage‐associated molecular pattern (DAMPs) released from the lysed bacteria and cancer cells recruit and activate immune cells, further enhancing immune cell infiltration through a positive feedback mechanism. This strategy reduces the side effects caused by bacteria and enhances anticancer activity.^159^

Cherenkov radiation‐induced photodynamic therapy (CR‐PDT) is an emerging form of PDT that does not require external light excitation. It utilises Cherenkov radiation generated by radioactive isotopes to activate nearby photosensitisers, leading to the generation of ROS that can damage target cells or tissues.^164^ Zhu et al. employed an aggregation‐induced emission photosensitiser (AIE‐PS) termed TTVP in combination with EcN to form a biological hybrid (EcN@TTVP). When EcN@TTVP, along with the radioactive drug ^18^F‐fluorodeoxyglucose (serving as the CR source), accumulates in the tumour site, it triggers CR‐PDT to produce ROS, inducing immunogenic cell death of tumour cells. Furthermore, EcN acts as an immunoadjuvant to enhance DC maturation and the activation of cytotoxic T lymphocytes (CTLs), enabling a synergistic effect between PDT and immunotherapy. Compared to single CR‐PDT, the AIE‐PS/bacterial hybrid system achieved efficient tumour regression and prolonged survival in tumour‐bearing mice (Figure 7B).^160^

### 5.2 Engineered bacteria for combination of TDT and immunotherapy

The modification of bacteria with thermosensitive materials and the regulation of temperature for activation have emerged as promising strategies. Ektate et al. developed a novel chemotherapy‐immunotherapy approach based on *S*. Typhimurium, where temperature‐sensitive liposomes were attached to the bacterial membrane to form thermobots. These thermobots deliver drugs to colorectal cancer cells and release DOX upon exposure to focused ultrasound heating (40–42°C), while simultaneously promoting the polarisation of macrophages to the M1 phenotype, enabling synergistic treatment of colorectal cancer (Figure 7C).^161^ Ma et al. used Fe_3_O_4_@lipid nanocomposites to construct a magnetic‐field‐controlled tumour‐homing bacterium. The engineered bacteria, controlled by a constant magnetic field, enhanced tumour targeting and improved therapeutic efficacy. The bacterial lysates, with their potent immunogenicity, synergised with anti‐CD47 nanobodies to activate both innate and adaptive immune responses. After accumulating in subcutaneous colorectal tumours in female mice, the paramagnetic Fe_3_O_4_ nanoparticles enabled the engineered bacteria to receive magnetic signals, which were converted into heat to trigger the expression of lysis proteins under the control of thermosensitive promoters. The engineered bacteria then lyse, releasing the anti‐CD47 nanobody cargo, thereby exerting potent anticancer effects through tumour‐specific CD47 blockade and precise tumour immunotherapy.^165^

Gene modification can be employed to construct engineered bacteria for thermally controlled drug release, which does not fall under the traditional definition of TDT, providing new insights for the future construction of engineered bacteria. Yang et al. chemically modified the surface of bacteria with DOX to develop ultrasound‐visible engineered bacteria. The engineered bacteria (Ec@DIG‐GV) can generate gas vesicles, which provide real‐time imaging guidance for remote hyperthermia high‐intensity focused ultrasound. Upon exposure to hHIFU, the temperature increases, activating the leftwards (pL) and rightwards (pR) phage lambda promoters to express the heat‐inducible IFN‐γ gene. This induces tumour cell death and promotes the polarisation of macrophages from the M2 to the M1 phenotype, while also enhancing DC maturation. DOX is released in the acidic TME, leading to immunogenic cell death of tumour cells. IFN‐γ and DOX can activate a tumour‐specific T‐cell response, leading to a stronger anti‐tumour effect.^166^ As our understanding of the interactions between bacteria and the TME continues to grow, it is likely that engineered bacteria will play an increasingly important role in the future of precision medicine.

### 5.3 Engineered bacteria for combination of SDT and immunotherapy

Engineered bacteria can be utilised for SDT in combination with immunotherapy. Ultrasound, a widely used clinical imaging modality known for its non‐invasive nature, lack of ionising radiation and excellent tissue penetration, is increasingly applied for precise tumour localisation and treatment.^167^ SDT relies on the accumulation of ultrasound‐sensitive agents at the tumour site, but current nanoparticle‐based therapies often face challenges related to tumour heterogeneity and poor targeting, leading to inadequate agent accumulation.^156^ In contrast, certain bacteria exhibit a natural ability to target the TME, making them ideal carriers for microenvironment‐directed delivery. Du et al. conjugated a nanosonosensitiser composed of poly (lactic‐co‐glycolic acid) (PLGA) nanodroplets (HPND), loaded with sonosensitiser haematoporphyrin monomethyl ether (HMME) and perfluoro‐n‐pentane (PFP), to the surface of EcN. EcN effectively transports the nanosonosensitiser to the tumour site, where ultrasound‐induced liquid‐gas phase transition of HPNDs@EcN occurs, leading to the release of the sonosensitiser HMME and the efficient generation of ROS, thereby exerting tumour‐killing effects. Mice with 4T1 mammary carcinoma show a significant reduction in tumour volume and a marked extension of survival time after treatment (Figure 7D).^162^

### 5.4 Engineered bacteria for PTT

PTT primarily utilises the thermal conversion effect of photothermal agents to raise the local temperature of tissues or cells above 40°C using specific wavelengths of light, thereby inducing cell death.^168^ Yin et al. constructed Bac@Au‐Ce6 by combining the photosensitiser Ce6 and gold nanoparticles (Au NPs) as photothermal agents with the photosynthetic bacteria *Synechococcus* 7942. Under 660 nm laser irradiation, Ce6 in Bac@Au‐Ce6 is activated to produce singlet oxygen. ROS interacts with biomolecules in tumour cells, causing damage and cell death. At the same time, the photosynthetic bacteria generate oxygen via photosynthesis, alleviating tumour hypoxia and enhancing the efficiency of PDT. Under 808 nm laser irradiation, the Au NPs in Bac@Au‐Ce6 absorb light energy and convert it to thermal energy, raising the temperature at the tumour site and further killing tumour cells. The combination of PDT and PTT significantly enhances therapeutic efficacy, and the bacteria can efficiently metabolise in major organs, ensuring substantial biosafety (Figure 7E).^163^ Chen et al. modified photothermal bacteria (PTB) with palladium nanoparticles (Pd NPs) to enhance their photothermal performance in the NIR region. They then synthesised a photosensitiser, methylene blue (MB), loaded onto ZIF‐90 (ZIF‐90/MB), which was covalently coupled to the PTB surface via acid‐sensitive imine bonds, forming PTB@ZIF‐90/MB. In the acidic tumour TME, ZIF‐90 degrades by ATP, releasing MB from the PTB surface and selectively delivering it to the mitochondria. Under NIR irradiation, MB generates singlet oxygen species, which disrupt mitochondrial redox homeostasis, impair mitochondrial function and ultimately inhibit ATP production. This downregulates heat shock proteins (HSPs) such as HSP70 and HSP90, weakening the tumour's resistance. Meanwhile, when Pd NPs on the bacterial surface absorb NIR light, they convert the light energy into heat, generating localised hyperthermia at the tumour site, thereby achieving effective PTT for tumours.^169^ These methods are advantageous because they can minimise damage to surrounding healthy tissues and can be precisely controlled by adjusting the intensity and duration of the light exposure. Engineered bacteria can be further modified to enhance their tumour‐targeting ability and to carry additional therapeutic agents, potentially improving the overall efficacy of the treatment.

### 5.5 Engineered bacteria for multi‐modal synergistic therapy

Different therapeutic approaches offer distinct mechanisms of tumour cell killing and have their respective advantages. Multi‐modal synergistic therapy, which combines multiple therapeutic strategies, is considered the optimal method to enhance therapeutic efficacy. Even wild‐type bacteria possess the potential for multi‐modal synergistic therapy. Non‐pathogenic natural purple photosynthetic bacteria (PPSB) have shown multifunctionality and biocompatibility in treating highly aggressive cancers. PPSB can convert NIR light into NIR fluorescence, thermal energy and ROS through an energy transfer system in its photosynthetic membrane nanocomplexes. This makes PPSB highly effective for tumour elimination and precise tumour localisation, with the assistance of the immune system. The combination of PPSB treatment and short‐term fasting induces an anti‐cancer immune response, significantly enhancing the optical efficacy of bacteria.^170^

Engineered bacteria are being employed for the precise targeting of tumour sites to facilitate multi‐modal synergistic treatment. Chen et al. employed PDA‐modified *Salmonella* VNP20009, a facultative anaerobic bacterium, as a PTT material. After intravenous injection of the bacteria, the tumour site was irradiated with NIR light. The pDA‐coated VNP20009 absorbed NIR light and converted it into heat, killing the cancer cells. In parallel, a phospholipid‐based phase separation gel containing anti‐PD‐1 peptide (AUNP‐12) was locally administered at the same site. The gel sustained the release of AUNP‐12 over 42 days, maintaining an immune‐permissive TME. The synergistic effect of biotherapy, PTT and sustained PD‐1 blockade therapy demonstrates significant advantages in the mouse melanoma model, resulting in a median survival time of 70 days for tumour‐bearing mice.^171^ Subsequently, the team chemically modified the surface of VNP20009 with newly synthesised heptamethine cyanine dyes NHS‐N782 and JQ‐1 derivatives, resulting in the biohybrid N‐V‐J. NHS‐N782 possesses mitochondrial targeting ability, and upon reaching the tumour, N‐V‐J is sensitive to temperature elevation and can eliminate the tumour via PTT triggered by NIR laser. The JQ‐1 derivative can be released from the bacterial surface, reversing the high expression of PD‐L1 in the TME and enhancing sustained immune responses. By activating N‐V‐J through NIR laser irradiation, tumour ablation is achieved, and immune responses are activated, converting the ‘cold’ TME into a ‘hot’ state. The intrinsic immunogenicity of bacteria, tumour antigens triggered by NIR, and the downregulation of PD‐L1 expression synergistically combine to effectively combat chronic tumours and their recurrence or metastasis through a multi‐strategy approach.^172^ Xie et al. developed such a system by loading the chemotherapeutic agent 5‐FU and the macrophage phenotype regulator zoledronic acid (ZOL) into EcN using electroporation. Subsequently, Au nanorods were modified onto the surface of EcN to construct EcN_Z/F_@Au. Under NIR illumination, the photothermal effect of AuNRs elevated the local temperature, triggering the in vivo transformation of EcN into bacterial ghosts (BGs). This transformation created transmembrane channels in the BGs, initiating gradual drug release. By applying intermittent NIR irradiation, the formation of BGs and drug release were progressively enhanced, allowing for external on‐demand control and spatiotemporal drug delivery. Within this system, FU exerted its chemotherapeutic effects through cytotoxic activity, while ZOL facilitated effective polarisation of TAMs to the M1 phenotype, promoting the production of pro‐inflammatory cytokines. This combination induced a synergistic effect, effectively inhibiting tumour growth through the integration of chemotherapy, immunotherapy and photothermal effects, with approximately 33% of 4T1 tumour‐bearing mice surviving for 52 days under EcN_Z/F_@Au/NIR treatment.^173^ The integration of these strategies not only improves the therapeutic outcome but also reduces systemic side effects, paving the way for personalised cancer therapy.

## 6 AI‐DRIVEN BACTERIA‐BASED CANCER THERAPY

AI refers to a broad field within computer science, which includes technologies like machine learning, deep learning, computer vision and natural language processing. These technologies enable machines to simulate human intelligence, allowing them to perform complex tasks. Machine learning and deep learning models rely on vast, diverse datasets for training, enabling these systems to identify patterns, make predictions and classify information based on the inputs they receive. In the future, AI will be extensively applied to novel bacteria‐based anti‐tumour therapies, primarily focusing on two key areas: AI‐enabled synthetic biology research and nanobiotechnology research.

### 6.1 AI‐enabled engineering of synthetic biology‐based microbial strains

The engineered bacteria modified through synthetic biology primarily activate immune responses or induce tumour cell killing by expressing specific substances. These processes are governed by complex genetic circuits, enabling controlled regulation, reducing toxic side effects and enhancing interactions with the host. Therefore, the future application of AI in this field is essential to reduce the labour‐intensive experimental work typically required and to improve the efficiency of developing bacteria‐based cancer therapies.

#### 6.1.1 AI‐driven promoter screening and optimisation

The selection of an appropriate promoter is a key aspect in the construction of engineered strains, as it enhances the expression of the target substance or makes its expression controllable. Designing promoters with desired characteristics is an important method to enhance the controllability of bacteria‐based cancer therapy. Deep learning can be used to predict the activity and gene expression effects of different promoters, thereby improving the precision regulation of synthetic biology systems. The properties of promoters are primarily determined by transcription factor binding sites (TFBS), and most researchers design new promoters by combining and arranging TFBS motifs. Wang et al. proposed a de novo promoter sequence design method based on deep learning. The trained generative adversarial networks (GAN) model can extract key features from natural promoter sequences and generate entirely new artificial sequences. The generated sequences successfully mimic the main features of natural promoters, including *k*‐mer frequency, motifs in the −10 and −35 regions, and their spacing constraints. After screening through a promoter activity prediction model, approximately 70.8% of AI‐generated promoters were experimentally validated as effective. Many AI‐designed promoters exhibited activity comparable to or even higher than that of most active natural promoters and their strongest mutants.^174^ The flanking sequences of TFBS also significantly affect promoter characteristics. Zhang et al. designed DeepSEED, which utilises a conditional GAN to generate flanking sequences based on predefined sequence elements, and uses another model based on DenseNet‐LSTM (with LSTM referring to ‘long–short‐term memory’) to predict promoter properties. DeepSEED has demonstrated outstanding effectiveness in tasks such as the design of prokaryotic constitutive promoters and prokaryotic IPTG‐inducible promoters, showing a high success rate.^175^

Conventional models may not achieve optimal performance and require consideration of multiple factors. It is necessary to incorporate various models and optimise them based on high‐throughput data integration, thereby enhancing the reliability of promoter screening. The screening of promoters using machine learning or deep learning methods often neglects evolutionary information, leading to unavoidable interference. Ren et al. utilised an improved DenseNet and Transformer, combined with a merged chaotic game representation, to propose a Chaotic Attention Network for Promoter Evolution (CAPE). By extracting evolutionary information from promoter sequences, this deep learning model achieved a Pearson correlation coefficient of .52 on the dataset through fivefold cross‐validation.^176^

AI can be leveraged to automate the design of promoters and optimise regulatory strategies by adapting the strains to different environmental conditions. For instance, *Halomonas* is a type of Gram‐negative, halophilic, alkaliphilic, rod‐shaped bacterium that can survive at a salt concentration of 60 g/L and a pH of 8–9. Its unique high‐salinity and high‐alkalinity survival conditions likely result in promoters with distinctive gene structures. To achieve more specific promoter screening, Zhao et al. developed a promoter strength database for the extremophilic microorganism *Halomonas* and proposed a novel promoter design and prediction method based on GANs and multi‐model fusion. They ultimately integrated deep learning (BiLSTM and convolutional neural network (CNN)) with traditional machine learning (random forests) to create a hybrid predictive model, enabling accurate selection of candidate promoters.^177^ In the future, AI could be applied to optimise the design of promoters responsive to the TME, including low oxygen, low pH, high lactate and high GSH concentrations.

#### 6.1.2 AI‐enabled gene circuit design and optimisation

AI‐assisted construction of complex gene expression network models facilitates the intelligent design and regulation of multi‐gene systems. Yang et al. utilised the flexible neural tree (FNT) model combined with genetic programming and simulated annealing optimisation algorithms, iteratively refining the structure and parameters of gene regulatory networks (GRNs) through evolutionary tree structures and parameter adjustments. The study employed a voting strategy and Akaike Information Criterion to identify the minimal set of regulatory genes, thereby enhancing the model's ability to accurately reconstruct GRNs and predict time‐series gene expression profiling. This highlights the application of AI in modelling complex biological data.^178^

AI can be used to optimise logic gate circuits in genetic networks, enabling precise regulation of gene functions. Merzbacher et al. applied Bayesian optimisation to learn the shape of the performance landscape and iteratively navigate the design space to identify the optimal circuit. This approach has broad applicability to genetic circuits, enabling the control of highly non‐linear biosynthetic pathways, assessing circuit robustness to perturbations, managing multiple interaction scales and achieving various performance goals.^179^ Saltepe et al. integrated LSTM neural networks with genetic circuits to significantly enhance the performance of whole‐cell biosensors (WCBs). By analysing sensor output data with the LSTM network, they successfully reduced response time and improved the prediction accuracy of gold ion concentrations, enabling the sensor to quickly and accurately detect the presence and concentration of gold ions. This AI integration approach holds promise for addressing the signal response issues in engineered bacteria.^180^

For more complex regulatory circuits, such as dynamic control of collective behaviours, AI can assist in optimising group collaboration and their responses to external stimuli, thereby enhancing therapeutic effects. Wu et al. used deep neural networks (DNN), support vector machines (SVMs), k‐nearest neighbours (KNN) and random forests to process and analyse large‐scale gut microbiome QS data from various sources. Their study classified the QS synthetases and receptors of gut microbes using these algorithms, uncovering numerous potential QS entries and constructing a comprehensive QS database (QSHGM). Building upon this, the study further expanded the database and identified novel QS proteins by comparing and analysing different datasets. These proteins are crucial for understanding microbial interactions and developing QS‐based microbial therapeutic methods.^181^ Rahman et al. developed DeepQSP for quorum sensing peptides (QSP) identification, which combines latent semantic analysis (LSA), a word embedding feature extraction method, with classical amino acid‐based pseudo amino acid composition and a CNN classifier. This is another pioneering study in QSP prediction, offering significant improvements in unravelling the complexities of QS.^182^

#### 6.1.3 AI‐based simulation of bacterial–host interactions

Based on AI‐driven host specificity assessment, the study by Lupolova et al. extracted genomic data and predicted protein variants (PVs) along with their annotations from *Salmonella enterica* and *E. coli*. These PVs served as input features for the machine learning model. By using the PVs from the genomes as features and combining them with host source information, an SVM classifier was trained. The SVM model learned the genomic characteristics of different host sources, enabling the establishment of a host specificity prediction model. The trained SVM classifier can accurately predict the host origin of *Salmonella* and *E. coli* isolates, which can be used for prevention or engineering applications.^183^ As large amounts of data on bacteria‐based cancer therapies are compiled, systems biology can be integrated with AI to explore the metabolic pathway interactions between bacteria and hosts. By leveraging the predictive capabilities of machine learning models, drug responses can be simulated by calibrating the pathway features of patients, thus optimising therapeutic strategies.^184^

### 6.2 AI‐enabled engineering of bacterial strains for nanomedicine applications

Currently, the application of AI in the development of bacteria‐based nanomedicines is still in the exploratory stage, primarily focusing on key aspects such as the design, optimisation and performance evaluation of nanodrugs. By analysing vast amounts of experimental data and literature, AI can predict the drug delivery efficiency, biocompatibility and therapeutic efficacy of different types of nanocarriers, thereby reducing development timelines and increasing success rates. In the dynamic monitoring and precise regulation of drug delivery systems, AI applications facilitate real‐time analysis and optimisation of the drug release process, further enhancing the clinical efficacy of bacteria‐based anticancer therapies. Nevertheless, the application of AI in this field remains far from fully mature and requires further exploration and practical application, particularly in the areas of interdisciplinary collaboration and the integration of large‐scale data.

#### 6.2.1 Construction of bacteria–nanoparticle composite systems

The application of machine learning techniques to optimise the targeting modification strategies of nanomaterials holds significant promise in enhancing the specificity of nanomedicines. Recent studies have already employed molecular dynamics and Monte Carlo simulations to predict and validate the interactions between drugs and nanomaterials, thereby improving the compatibility of the materials with the biological system. This approach has shown potential in refining the design of nanocarriers to achieve better drug delivery efficiency and reduced off‐target effects.^185^ A growing body of research is focusing on computational simulations of bacterial surface structures, with the aim of understanding and manipulating the bacterial exterior for therapeutic purposes.^186^ Ain et al. innovatively and collaboratively utilised AI and nanotechnology to develop a novel class of nanosynbiotics. AI was employed for formulation design and optimisation, as well as biological activity prediction. The AI‐driven nanosynbiotics possess efficient delivery, targeted regulation and safety features.^187^ AI can assist in molecular dynamics simulations of interface interactions, optimising the interaction between bacteria and nanoparticles. By constructing accurate models of bacterial structures, AI can leverage high‐throughput computations and simulations to rapidly identify effective candidate materials and drug molecules, enabling the optimisation of compatibility in bacteria‐based anticancer drug development. The integration of AI with these computational tools could ultimately lead to more efficient, personalised and targeted cancer treatments, maximising therapeutic outcomes while minimising side effects.

#### 6.2.2 Intelligent planning and monitoring of in vivo delivery pathways

Engineered bacteria are increasingly utilised as vehicles for the targeted delivery of anticancer drugs or active therapeutic materials.^188^ To enhance the therapeutic efficacy and minimise potential side effects, it is essential to optimise the pharmacokinetics of these drug delivery systems within the body. Constructing AI‐based multiscale pharmacokinetic models plays a pivotal role in achieving this optimisation. These models enable the precise prediction of drug distribution, absorption, metabolism and clearance across various biological scales, from cellular interactions to whole‐body dynamics.^189^ By leveraging AI, such models can simulate and analyse complex biological processes, identify key factors influencing drug behaviour and refine strategies to improve drug delivery efficiency and therapeutic outcomes.

The advantage of bacteria‐based delivery systems lies in their excellent ability to target and colonise tumours. However, these systems must overcome various physiological barriers, and their ability to traverse biological barriers significantly impacts their distribution and pharmacological effects in vivo, directly influencing the drug's efficacy and safety.^190^, ^191^ Recent studies have demonstrated the use of AI to analyse the delivery of nanoparticle‐based drugs. AI strategies that predict the ability of engineered bacteria to penetrate the blood‐brain barrier, cell membranes and visceral barriers may offer new directions to enhance the effectiveness of bacteria‐based drug delivery systems.^192^

Several studies have employed AI to track particles dynamically within living cells, facilitating the complex analysis of 3D cargo transport processes. This advancement has opened up new possibilities for understanding cellular dynamics in real‐time.^193^ With the ongoing development of bacterial delivery systems, novel engineered bacteria that are capable of dynamic tracking have been introduced. These engineered strains present promising potential for more accurate and targeted delivery of therapeutic agents.^194^ The integration of these technologies will enable precise control over microenvironmental targeting, predicting and tracking the behaviour of engineered bacteria in vivo, thereby improving the efficiency of drug delivery systems and minimising adverse side effects. This advancement could significantly enhance therapeutic outcomes and the safety profiles of engineered bacteria‐based delivery systems in clinical applications.

A major development direction for tumour‐targeting engineered bacteria based on techniques such as photothermal and ultrasound methods is the controlled release of drugs. Therefore, developing predictive models for drug release is crucial. For instance, research has already utilised artificial neural network models to simulate and perform sensitivity analysis on the acoustic release of DOX from the unstable Pluronic P105. Accurate simulation of drug release and optimisation of release conditions are essential for improving the therapeutic efficacy of engineered bacteria. By refining the prediction models and understanding the underlying mechanisms of drug delivery, it is possible to enhance the precision of therapeutic interventions and optimise the overall effectiveness of bacteria‐based drug delivery systems in cancer treatment.^195^

### 6.3 AI‐based approaches for toxicity assessment and optimisation of pharmacological interventions

Bacterial drug susceptibility is closely related to its toxicity, with most bacteria causing harm to the host. Therefore, the selection of an appropriate chassis bacterium is crucial.^196^ The development of high‐throughput AI models to predict the immunogenicity of bacteria is essential for identifying suitable candidates from a vast array of bacterial species. These bacteria must exhibit targeted properties within the TME while minimising harm to the host organism. Such an approach will accelerate the drug development process by enabling the identification of bacteria that can effectively target tumours without inducing systemic damage.^197^, ^198^

A substantial amount of research has already been conducted utilising AI for drug toxicity prediction. These studies have employed various machine learning models, particularly focusing on the ability of AI to assess and predict the potential harmful effects of new compounds before they undergo clinical trials.^199^, ^200^ With advancements in data analytics and AI technologies, these predictive models have become more accurate, enabling the identification of toxicological risks at earlier stages of drug development.^201^ To accelerate the development of bacteria‐based cancer therapeutics, employing deep learning frameworks for toxicity assessment will be an essential strategy.

AI can be utilised to assess patient conditions and optimise future drug usage. For instance, fuzzy logic can be applied to develop knowledge‐based decision algorithms, which analyse a patient's hemodynamic status, including parameters such as cardiac index, systemic vascular resistance index and pulmonary vascular resistance index, to evaluate the patient's health condition. Based on this analysis, the system can recommend the most appropriate drug treatment plan for different situations. This approach allows the system to handle various cases and optimise drug usage, leading to more precise treatment adjustments.^202^

## 7 CONCLUSIONS AND PROSPECTS

Bacteria‐based active materials for precision cancer diagnosis and therapy have demonstrated immense potential and broad application prospects. The unique tumour‐targeting ability, biocompatibility and controllability of bacteria make them ideal candidates for drug delivery vehicles and immune modulators. With the continuous development of synthetic biology and nanomodification technologies, bacterium‐based cancer therapies are becoming increasingly diversified.

Through genetic modification, bacteria can be modified to express specific tumour‐targeting peptides, receptors or signalling molecules, enhancing their targeting ability. In addition, bacteria can serve as platforms to combine multiple therapeutic approaches, such as chemotherapy, PDT and PTT, achieving synergistic effects and improving treatment outcomes. However, maximising the reduction of side effects and translating these therapies into clinical practice still presents significant challenges. During the design process, it is essential to clarify the mechanisms of action and continuously optimise and screen the bacteria through genetic engineering and other approaches to identify the most suitable engineered strains.

Bacterial therapy holds tremendous potential in cancer treatment, offering new strategies for highly precise tumour targeting with minimal side effects. Recent advancements in genetic engineering and synthetic biology have paved the way for the development of complex bacterial systems capable of directly delivering a wide range of therapeutic agents to cancer cells. In the future, bacteria‐based tumour therapy should increasingly focus on multi‐effect synergistic treatments to maximise therapeutic efficacy and minimise side effects, with AI playing a pivotal role in advancing such therapies, particularly in the fields of synthetic biology and nanobiotechnology. These innovations will likely include AI‐driven designs for optimised bacterial genomes, metabolic pathways and autonomous behaviours in synthetic biology, enabling bacteria to target tumours more effectively. In nanobiotechnology, AI will facilitate the development of intelligent nanocarriers for precise drug delivery and multi‐modal therapeutic platforms that integrate diagnosis and treatment. Furthermore, the integration of synthetic biology and nanobiotechnology, empowered by AI, will pave the way for autonomous treatment systems capable of real‐time monitoring and dynamic regulation of therapeutic responses. While challenges such as data integration, algorithm interpretability and clinical translation remain, the potential for AI to revolutionise bacteria‐based cancer therapies is immense, promising more efficient, precise and safer treatment solutions.

## AUTHOR CONTRIBUTIONS

*Conceptualisation*: Shuai Fan and Siyu Zhu. *Investigation*: Shuai Fan, Siyu Zhu and Wenyu Wang. *Writing—original draft preparation*: Shuai Fan and Siyu Zhu. *Writing—review and editing*: Shuai Fan, Siyu Zhu and Wenyu Wang. *Funding acquisition*: Shuai Fan, Hao Li, Wenyu Wang, Lei Dong and Qin Xia. *Visualisation*: Yuetong Liu, Yutong Zhou, Shuai Fan and Siyu Zhu. *Supervision*: Lei Dong, Qin Xia and Bofeng Liu. All the authors have read and agreed to the published version of the manuscript.

## CONFLICT OF INTEREST STATEMENT

The authors declare no conflicts of interest.

## ETHICS STATEMENT

This study did not involve human or animal subjects, and thus, no ethical approval was required.

## CONSENT

Not applicable.

## Data Availability

All data generated or analysed during this study are included in this published article.
